# Transcriptomic characterization of platelet-rich fibrin-induced macrophage responses identifies U937 cells as a sensitive bioassay

**DOI:** 10.3389/fimmu.2026.1722342

**Published:** 2026-04-22

**Authors:** Layla Panahipour, Xiaoyu Huang, Francesca Zampino, Richard J. Miron, Reinhard Gruber

**Affiliations:** 1 Department of Oral Biology, University Clinic of Dentistry, Medical University of Vienna, Vienna, Austria; 2 Department of Prosthodontics, The Affiliated Stomatological Hospital of Southwest Medical University, Luzhou, China; 3 Department of Periodontology, School of Dental Medicine, University of Bern, Bern, Switzerland; 4 Austrian Cluster for Tissue Regeneration, Vienna, Austria

**Keywords:** bulk RNA sequencing, macrophage polarisation, platelet-rich fibrin, THP1, U937

## Abstract

Platelet-rich fibrin (PRF) is extensively utilized to enhance localized tissue healing, a process that critically depends on the transient polarization of macrophages toward a pro-inflammatory phenotype. Given that PRF, like other blood clot derivatives, may intrinsically modulate macrophage behavior, we conducted a comprehensive screening assay to characterize the global macrophage response to PRF exposure. To this end, we employed two widely used monocytic cell lines—U937 (histiocytic lymphoma) and THP-1 (acute monocytic leukemia)—as *in vitro* models to investigate macrophage responses. Cells were exposed to lysates derived from PRF, and transcriptomic alterations were profiled using bulk RNA sequencing. Differential gene expression analysis was performed, with significance determined by an adjusted p-value threshold of <0.05. In U937-derived macrophages, gene expression profiling revealed a transcriptional signature consistent with inflammatory activation. Clustering of upregulated genes highlighted pathways associated with chemokine activity (e.g., CCL2, CCL3, CCL4, CCL5, CCL7, CCL8, CCL20, CCL23, CCL26, CXCL5, CXCL6, CXCL8, CXCL16, and PPBP), RAGE receptor binding (FPR1, S100A8, S100A9, and S100A12), IgG binding (FCGR1A, FCGR2A, FCGR2B, and FCGR3A), prostaglandin biosynthesis (CBR1, CD74, EDN1, FABP5, IL1B, MIF, PTGES, and PTGS1), and collagen catabolism (CTSL, FAP, MMP3, MMP7, MMP9, MMP12, MMP14, MMP19, and MRC2). In contrast, PRF exposure in THP-1 cells primarily enriched genes involved in steroid biosynthesis, suggesting a more limited or distinct response. These findings underscore U937 cells as a more responsive and appropriate bioassay for modeling inflammatory macrophage polarization in response to PRF. Moreover, the identified gene signatures recapitulate key aspects of early wound healing, providing a relevant platform for studying macrophage reactivation in chronic wound environments.

## Background

Regenerative medicine has emerged as an umbrella discipline that integrates diverse scientific approaches to develop and optimize therapeutic strategies for impaired wound and bone healing, with expanding applications in dentistry (1). These regenerative interventions typically act locally to enhance the intrinsic healing cascade, most commonly through the delivery of growth factors (2, 3) and other bioactive molecules (4) within a three-dimensional matrix framework (5). Conceptually, these approaches are inspired by the evolutionarily conserved process of blood coagulation, wherein monocytes, platelets, and other blood cells become entrapped within a provisional fibrin-rich scaffold that later matures into vascularized granulation tissue, collectively referred to as a hematoma (6). During this early reparative phase, infiltrating monocytes and macrophages secrete a spectrum of pro-inflammatory cytokines and chemokines, establishing the hematoma as a transient yet dynamic immunomodulatory niche (7, 8). The tight evolutionary coupling of coagulation and inflammation underscores a conserved biological axis (9). The question of how blood-clot constituents mechanistically activate macrophages is particularly compelling.

Within a freshly formed blood clot, resident monocytes—and macrophages that infiltrate at later stages—are exposed to a decadent milieu of bioactive mediators released upon platelet activation, along with other components of the coagulum. This environment has prompted an intensive investigation into platelet–monocyte interactions as central determinants of thrombosis and inflammation (10). Indeed, direct contact between platelets and monocytes has been shown to induce a pro-inflammatory phenotype in circulating monocytes (11). Moreover, platelet-derived extracellular vesicles amplify this inflammatory response by upregulating pro-inflammatory cytokine expression in both monocytes (12) and synovial fibroblasts (13). Clinical observations further substantiate these findings: single-cell RNA sequencing of healing skin wounds has identified two macrophage clusters exhibiting pro-inflammatory transcriptional profiles during the early phase of repair (14). However, the molecular cues driving macrophage polarization toward this pro-inflammatory state remain incompletely understood. Elucidating how specific components of the blood clot modulate macrophage activation, therefore, captures a physiologically and clinically relevant paradigm—one that encompasses both natural tissue repair and therapeutic strategies employing autologous blood products to enhance regeneration. Collectively, these insights underscore a fundamental question central to regenerative biology: how do the constituents of a blood clot orchestrate macrophage polarization during the early stages of healing?

Regenerative medicine has leveraged the innate biological principles of the natural blood clot, transforming this evolutionary concept into fibrin-rich scaffolds filled with activated platelets and peripheral blood mononuclear cells (PBMCs). This has led to autologous preparations such as platelet-rich fibrin (PRF) (15), and when anticoagulants are used, platelet-rich plasma (PRP) (16). PRF is the coagulated plasma portion of centrifuged blood (17), marked by a high concentration of platelets and leukocytes while being largely free of erythrocytes (15). The idea of using a patient’s own plasma fraction to create a solid PRF membrane has gained widespread clinical acceptance, beginning predominantly in dental applications (15). Increasing clinical evidence supports the use of PRF for regenerative treatments of periodontal intrabony (18) and furcation defects (19), socket healing after third molar extraction (20), and alveolar ridge preservation (21). Remarkably, PRF has also shown significant therapeutic benefits for chronic wounds, including diabetic and venous ulcers (22). Beyond oral and skin applications, both PRF and PRP have been broadly adopted worldwide in facial aesthetics (11) as well as in orthopedic and sports medicine procedures (23, 24). Despite these encouraging clinical results, a significant knowledge gap remains in understanding the cellular mechanisms involved—especially the processes of macrophage activation and polarization in response to PRF.

Macrophages possess pleiotropic functions that extend far beyond their canonical role in host defense. They are now recognized as indispensable regulators of wound healing (25–28) and key contributors to bone regeneration (29, 30). These insights have positioned macrophages at the center of regenerative medicine research, prompting investigations into how PRF modulates their activity. Notably, PRF lysates have been reported to attenuate lipopolysaccharide (LPS)-induced inflammation in murine RAW 264.7 macrophages (31–33). However, these findings contrast with evidence from human studies, where platelet–monocyte interactions trigger a pronounced pro-inflammatory response (11, 12), and transcriptomic analyses reveal similar inflammatory signatures in PBMCs exposed to PRF lysates (34). Furthermore, substantial interspecies differences in wound-healing responses have been documented, exemplified by divergent S100A gene expression patterns between human and murine macrophages (14). A similar discrepancy is observed in fibroblasts, where PRF elicits a pro-inflammatory response in human cells (35) but an anti-inflammatory effect in murine counterparts (36). Collectively, these observations suggest that PRF may likewise drive macrophage polarization toward a pro-inflammatory phenotype in human systems. This hypothesis underscores the importance of species-specific investigation when translating preclinical findings into clinical applications.

The previously observed pro-inflammatory response of PBMCs exposed to PRF lysates (34) provides a compelling basis for investigating the mechanisms underlying PRF effects on macrophages. To this end, we used two widely established human monocytic cell lines—U937, derived from a histiocytic lymphoma (37), and THP-1, originating from an acute monocytic leukemia (38). Despite their neoplastic origin, both U937 and THP-1 cells are widely used in inflammation research because of their reproducible, well-characterized responses (39–41). Recent bulk RNA sequencing analyses have further clarified the complex transcriptional dynamics of these models when stimulated with LPS and IFNG (Interferon-gamma) (42). However, these cell lines have not yet been systematically validated as bioassays for PRF research. This study, therefore, aims to characterize the transcriptional response of macrophages derived from U937 and THP-1 cells to PRF lysates using RNA sequencing. Our goal is to identify a set of reliably regulated genes that could serve as sensitive molecular markers for assessing the biological activity of different PRF formulations and, more broadly, to explore how natural blood-clot components affect cells.

## Materials and methods

### Preparation of PRF membrane lysates

The preparation of platelet-rich fibrin (PRF) was approved by the Ethics Committee of the Medical University of Vienna (EK #1644/2018) and was performed in accordance with the Declaration of Helsinki and the principles of Good Clinical Practice. Venous blood was collected from three healthy adult donors and centrifuged at 700 × g for 8 min using a swing-out rotor centrifuge (Z306 Hermle, Universal Centrifuge, Wehingen, Germany) in glass tubes (Bio-PRF, Venice, FL, USA) to obtain PRF membranes. The yellow PRF clot was carefully separated from the residual red thrombus and gently compressed between two metal plates to form solid PRF membranes. These membranes were subsequently immersed in serum-free DMEM (1 cm PRF membrane per 1 mL medium) and subjected to two freeze–thaw cycles, followed by sonication (Sonopuls 2000.2, Bandelin Electronic, Berlin, Germany). The resulting PRF lysates were collected as supernatants after centrifugation of the disrupted membranes at 15,000 × g for 10 min (Eppendorf, Hamburg, Germany), aliquoted, and stored at –80 °C until further use. This study did not constitute a clinical trial, as PRF membranes were prepared exclusively for *in vitro* experimental purposes.

### U937 and THP-1

The human monocytic cell lines U937 (CRL-1593.2) and THP-1 (TIB-202) were obtained from the American Type Culture Collection (ATCC, Manassas, VA, USA). Cells were cultured in RPMI 1640 medium (Gibco, Life Technologies, Carlsbad, CA, USA) supplemented with 10% fetal calf serum (FCS; Bio&Sell GmbH, Nuremberg, Germany) and 1% penicillin–streptomycin, and maintained in a humidified incubator at 37 °C with 5% CO_2_. For macrophage differentiation, both cell lines were seeded into 24-well plates and stimulated with 10 ng/mL phorbol 12-myristate 13-acetate (PMA; Sigma-Aldrich, St. Louis, MO, USA) for 24 h. Differentiated U937- and THP-1-derived macrophages were subsequently exposed to 30% PRF lysate or serum-free medium (control) for 48 h under standard culture conditions (37 °C, 5% CO_2_, 95% humidity) before RNA extraction.

### Total RNA isolation, sequencing, and data analysis

Total RNA was isolated with the GeneMATRIX Universal RNA purification kit with DNase digestion (EUR_X_, Gdańsk, Poland). Sequencing libraries from total RNA were prepared at the Core Facility Genomics, Medical University of Vienna, using the QuantSeq 3’ FWD protocol version 2 with unique dual indices (Lexogen GmbH, Vienna, Austria). Fifteen PCR cycles were used for library preparation, as determined by quantitative PCR (qPCR) according to the library preparation manual. Libraries were QC-checked on a Bioanalyzer 2100 (Agilent Technologies, Santa Clara, CA) using a high-sensitivity DNA Kit to confirm correct insert size and quantified using the Qubit dsDNA HS Assay (Invitrogen, Waltham, MA). Pooled libraries were sequenced on a P2 flowcell on a NextSeq2000 instrument (Illumina, San Diego, CA) in 1x75bp single-end sequencing mode. On average, 7 million reads were generated per sample. Reads in FASTQ format were generated using the Illumina bcl2fastq command-line tool (v2.19.1.403) and the Lexogen idemux tool for optimal demultiplexing of long, unique, dual indices. Reads were trimmed and filtered using cutadapt version 2.8 to trim polyA tails, remove reads with N’s, and trim bases with a quality of less than 30 from the 3’ ends of the reads (43). On average, 5 million reads were left after this procedure. Trimmed reads in fastq format were aligned to the human reference genome version GRCh38 with Gencode 29 annotations using the STAR aligner (44) version 2.6.1a in 2-pass mode. STAR counted raw reads per gene. Differential gene expression was calculated using DESeq2 (45) version 1.22.2. The fastq files are available in the GEO repository under the accession number GSE308204.

### Reverse transcription quantitative real-time PCR

Total RNA was transcribed into complementary DNA (LabQ, Labconsulting, Vienna, Austria). Polymerase chain reaction was performed (LabQ, Labconsulting, Vienna, Austria) on a CFX Connect™ Real-Time PCR Detection System (Bio-Rad Laboratories, Hercules, CA). The primer sequences are in the **Supplementary Material** (**Supplementary Table 1**). The relative expression of each specific mRNA was normalized to the housekeeping gene GAPDH by the ΔΔCt method. Unstimulated controls are expressed as 1.0 in all analyses.

### Volcano plot, venn diagram, heat map, protein-protein interactions, and gene set enrichment analysis

Volcano plots were generated using VolcaNoseR, an open-access, web-based tool designed for the interactive visualization of differential expression analyses. This platform enabled the identification and graphical representation of genes that were significantly up- or downregulated based on log_2_ fold-change and adjusted p-value thresholds (46). Genes exhibiting at least a eight change in expression (|log_2_ fold change| ≥ 3.0) and a p-value ≤ 0.01 were considered significantly differentially expressed and selected for subsequent analyses (46). A comparative analysis of gene sets was performed using InteractiVenn, an interactive web-based tool that generates Venn diagrams to visualize shared and unique genes across datasets (47). Heatmaps were generated in R (v4.5.2; r-project.org) based on genes with an adjusted p-value < 0.05. Functional enrichment analysis was conducted using g:Profiler, a comprehensive web-based platform that integrates multiple annotation resources, including Gene Ontology, KEGG, Reactome, and other pathway databases, to identify overrepresented biological processes among differentially expressed genes (48).

## Results

### Principal component analysis (PCA) of gene expression changes by activated U937 and THP-1

**Figure 1:**
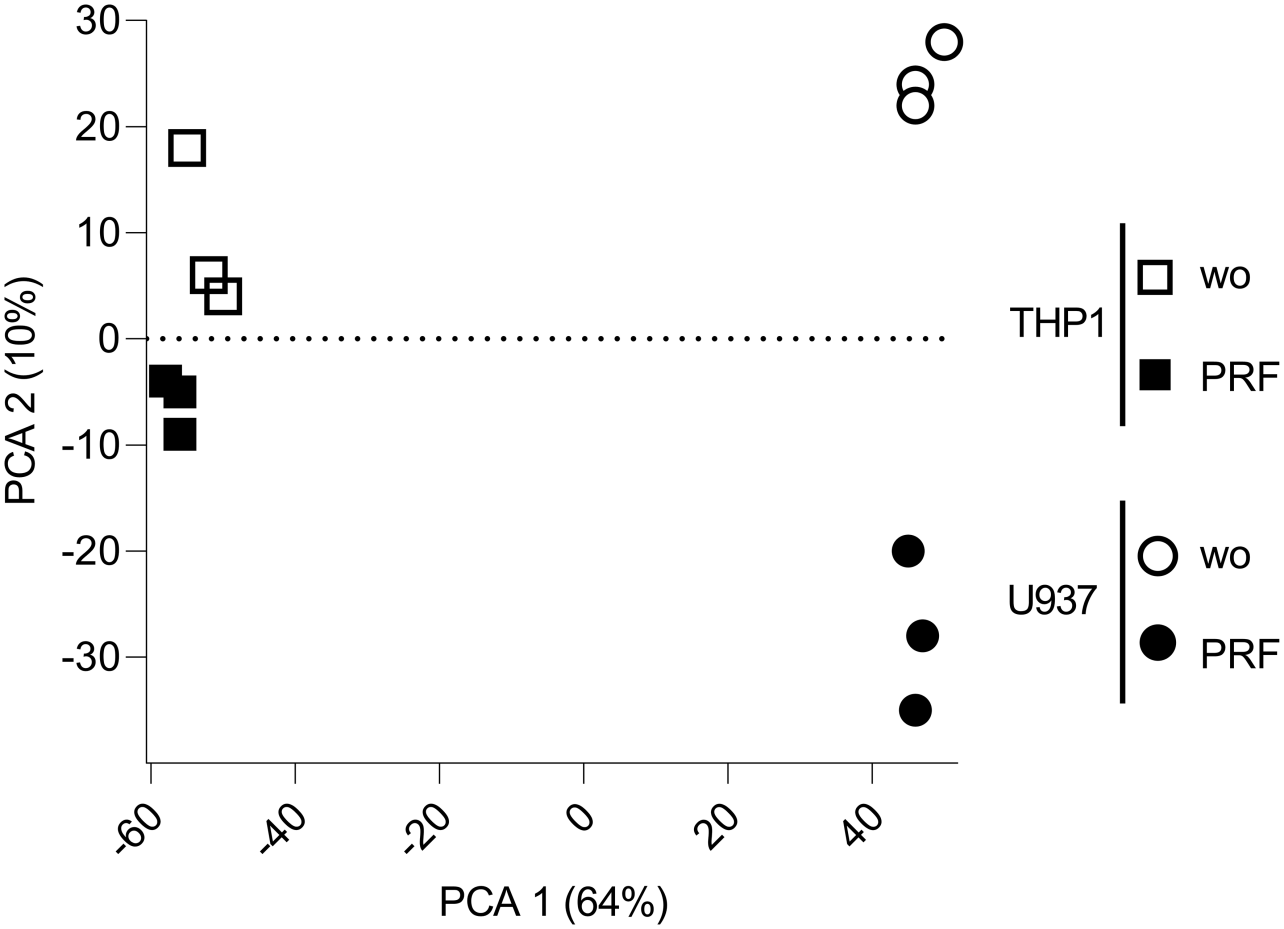
PCA,principal component analysis of differentially expressed genes in PRF-activated U937 and THP-1 macrophages. The plot depicts the sample distribution in a two-dimensional space defined by the first and second principal components of the covariance matrix. Gene expression values were normalized and expressed as logCPM, log counts per million.

To investigate the global transcriptional impact of PRF on macrophage-like cells, we performed bulk RNA sequencing of U937- and THP-1–derived macrophages exposed to PRF lysates (**Figure 1**). Principal component analysis (PCA) revealed that the first principal component captured most of the variance between the two cell types, reflecting their distinct transcriptional identities. The second principal component captured the gene expression changes induced by PRF exposure, which were more homogeneous but less pronounced in U937 cells compared to THP-1 cells (**Figure 1**). These findings indicate that while both models respond transcriptionally to PRF stimulation, the magnitude and variability of the response differ substantially between the two cell lines.

### Volcano analysis of gene expression changes by PRF-exposed U937 and THP-1 cells

**Figure 2:**
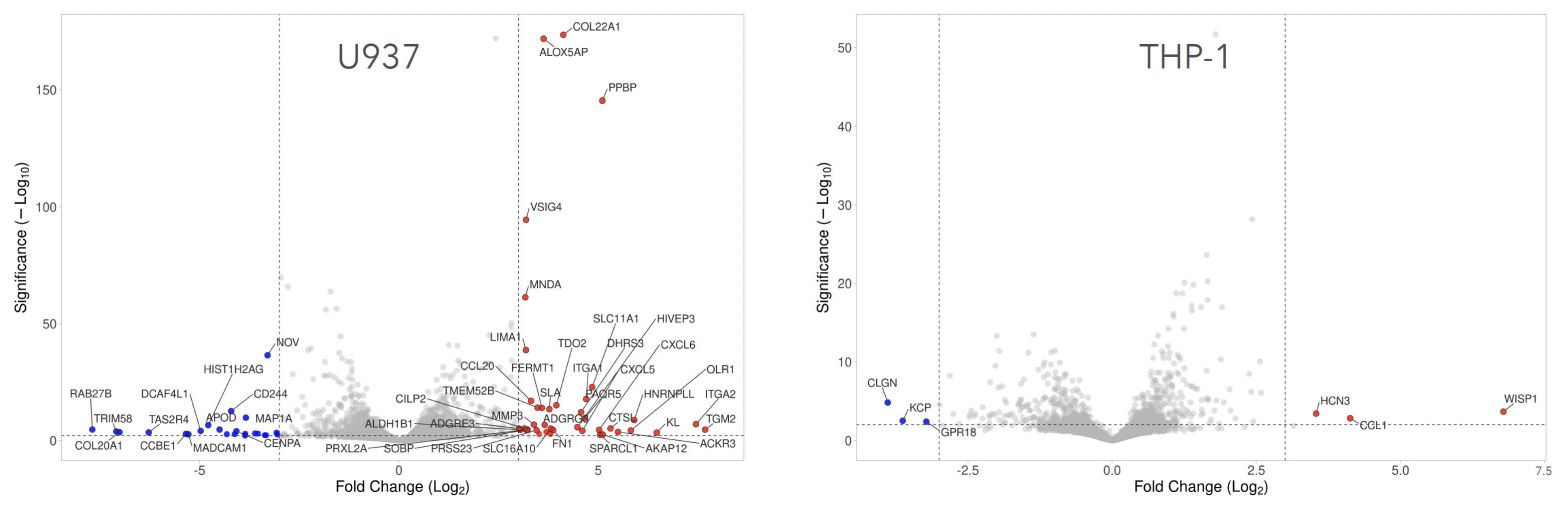
Volcano plot analysis of differentially expressed genes in U937 and THP-1 macrophages treated with PRF. Volcano plots illustrate upregulated (red) and downregulated (blue) genes in U937 and THP-1 cells following PRF exposure. The annotated data points represent the 50 genes with the greatest Euclidean (Manhattan) distance from the origin that exceed the defined significance thresholds (dashed lines). Thresholds were set at –log_10_ (p) ≥ 2.0 and |log_2_ fold change| ≥ 3.0.

To visualize the direction and magnitude of differential gene expression, we generated volcano plots (**Supplementary File 1**). Differentially expressed genes were defined using a combined statistical threshold of –log_10_ (p) ≥ 2.0 and |log_2_ fold change| ≥ 3.0. Based on these criteria, we identified 40 upregulated genes in U937 cells and 3 upregulated genes in THP-1 cells following PRF exposure. In U937 cells, the most strongly induced genes included chemokines (CCL20, CXCL5, CXCL6), adhesion G protein–coupled receptors (ADGRE3, ADGRG3), integrin α-subunits (ITGA1, ITGA2), as well as fibronectin (FN1), matrix metalloproteinase 3 (MMP3), and oxidized LDL receptor 1 (OLR1). In contrast, the most prominent transcriptional increase was observed in THP-1 cells for CCL1. Furthermore, 23 downregulated genes were detected in U937 cells and 3 in THP-1 cells (**Figure 2**). Among these, CENPA and CENPU were notably decreased in U937 cells, whereas in THP-1 cells, downregulation was restricted to CLGN, KCP, and GRP18.

### Heat map of gene expression changes in U937 and THP-1 cells by PRF lysates

**Figure 3:**
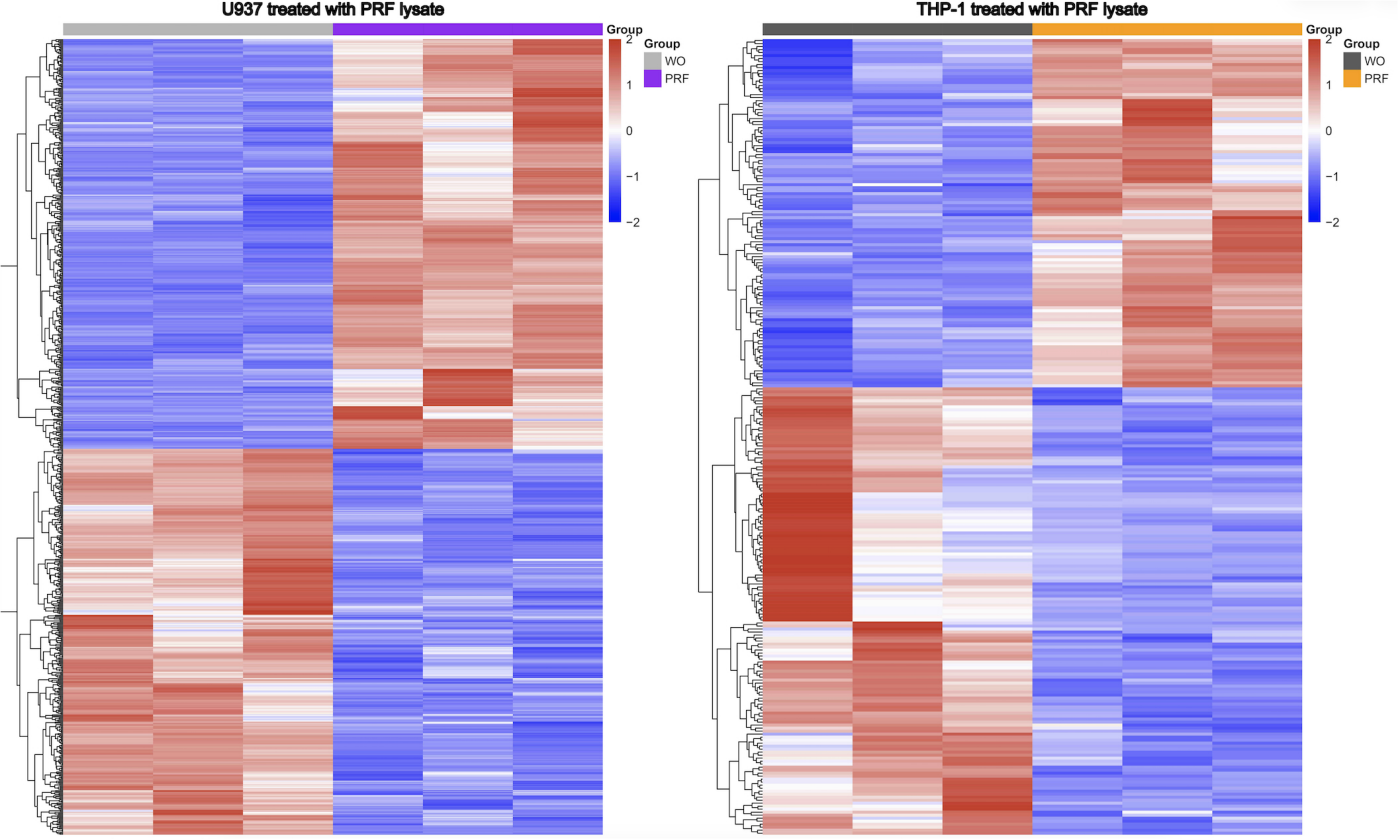
Heatmap of differentially expressed genes in U937 and THP-1 macrophages exposed to PRF lysates. The heatmap visualizes the 395 upregulated and 372 downregulated genes in U937 cells, and the 116 upregulated and 149 downregulated genes in THP-1 cells, following exposure to PRF lysates. Differentially expressed genes were defined by an adjusted p-value < 0.05. The dataset integrates transcriptional data from three independent PRF preparations. Color gradients indicate relative expression levels, with red representing upregulation and blue representing downregulation; color intensity reflects the magnitude of the expression change.

To apply a less stringent selection criterion than that used for the volcano plot, we generated heatmaps based on all significantly regulated genes with an adjusted p-value < 0.05 (**Supplementary File 2**). Under this threshold, 395 upregulated and 372 downregulated genes were identified in U937 cells, whereas 116 upregulated and 149 downregulated genes were detected in THP-1 cells (**Figure 3**). The resulting heatmap illustrates the global transcriptional response of both cell types to PRF lysates, revealing that U937 cells exhibit markedly stronger transcriptional modulation than THP-1 cells, indicating greater sensitivity of U937 macrophages to PRF stimulation.

### Venn analysis of genes regulated by PRF lysates in U937 and THP-1

**Figure 4:**
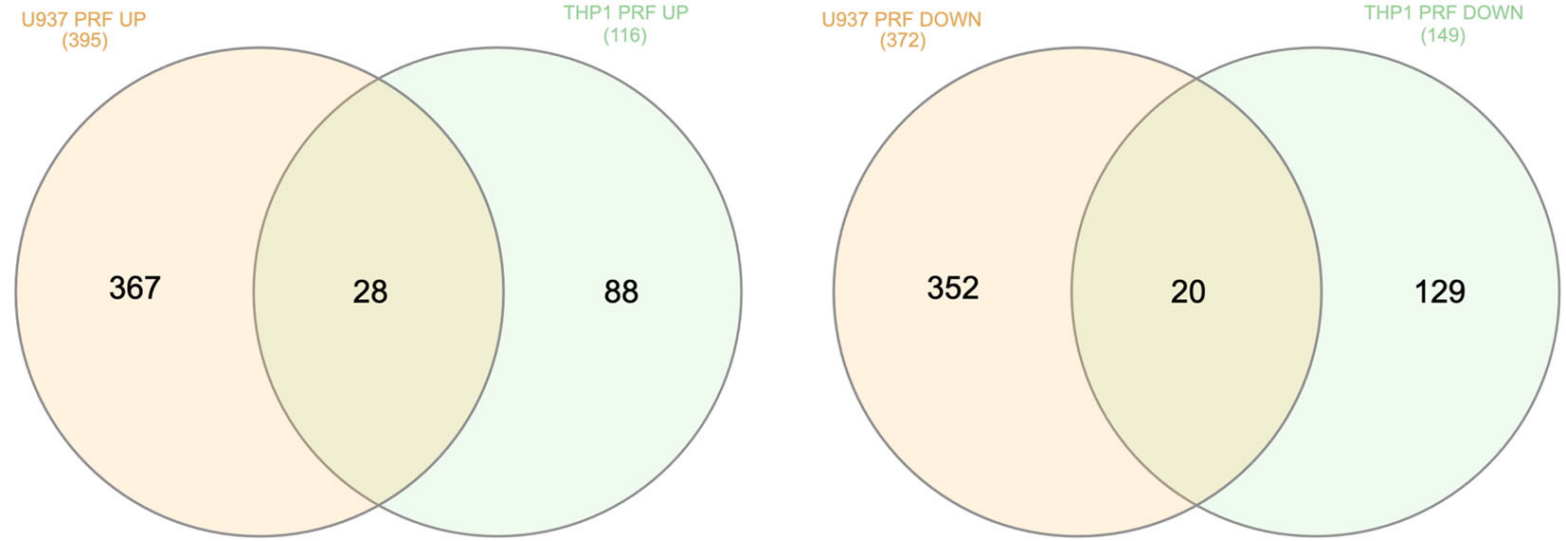
Venn analysis of differentially expressed genes in U937 and THP-1 macrophages treated with PRF lysates. Venn diagrams show the overlap of upregulated and downregulated genes between U937 and THP-1 cells after exposure to PRF lysates (adjusted p < 0.05). A total of 28 genes were commonly upregulated, and 20 genes were commonly downregulated in both cell lines.

To further delineate similarities and differences in the transcriptional responses of U937 and THP-1 macrophages to PRF lysates, we performed a Venn analysis of genes with adjusted p-values < 0.05. This comparison revealed 28 commonly upregulated and 20 commonly downregulated genes shared between the two cell lines (**Figure 4**), indicating that most transcriptional changes were cell–type–specific. Among the shared upregulated genes were FN1, ITGA6, LIF, MMP7, NFATC2, OSM, S100A12, SERPINA1, TGM2, and TLR2—none of which are canonical chemokines associated with an inflammatory macrophage phenotype. The commonly downregulated genes included CD48, CXCR4, and MMP25. Overall, the set of shared genes did not exhibit a distinct functional pattern, underscoring differential, cell-line–specific responses of U937 and THP-1 macrophages to PRF lysate stimulation (**Supplementary File 3**).

### G: profiler analysis of gene expression changes in U937 and THP-1 cells by PRF lysate

To further explore the biological pathways underlying the transcriptional response to PRF, we performed functional enrichment analysis of differentially expressed genes in U937 macrophages (adjusted p < 0.05; **Supplementary File 4**). Gene Ontology (GO) analysis revealed strong enrichment in molecular function (MF) categories associated with chemokine activity (GO:0008009), including CCL2, CCL3, CCL4, CCL5, CCL7, CCL8, CCL20, CCL23, CCL26, CXCL5, CXCL6, CXCL8, CXCL16, and PPBP, indicating a pronounced chemokine-driven inflammatory signature induced by PRF lysates.

Additional enrichment was observed in RAGE receptor binding (GO:0050786), comprising FPR1, S100A8, S100A9, and S100A12, and in IgG binding (GO:0019864), involving FCGR1A, FCGR2A, FCGR2B, and FCGR3A—consistent with an overall proinflammatory macrophage phenotype. Biological process (BP) categories further supported this interpretation, notably prostaglandin biosynthetic process (GO:0001516), represented by CBR1, CD74, EDN1, FABP5, IL1B, MIF, PTGES, and PTGS1.

**Figure 5:**
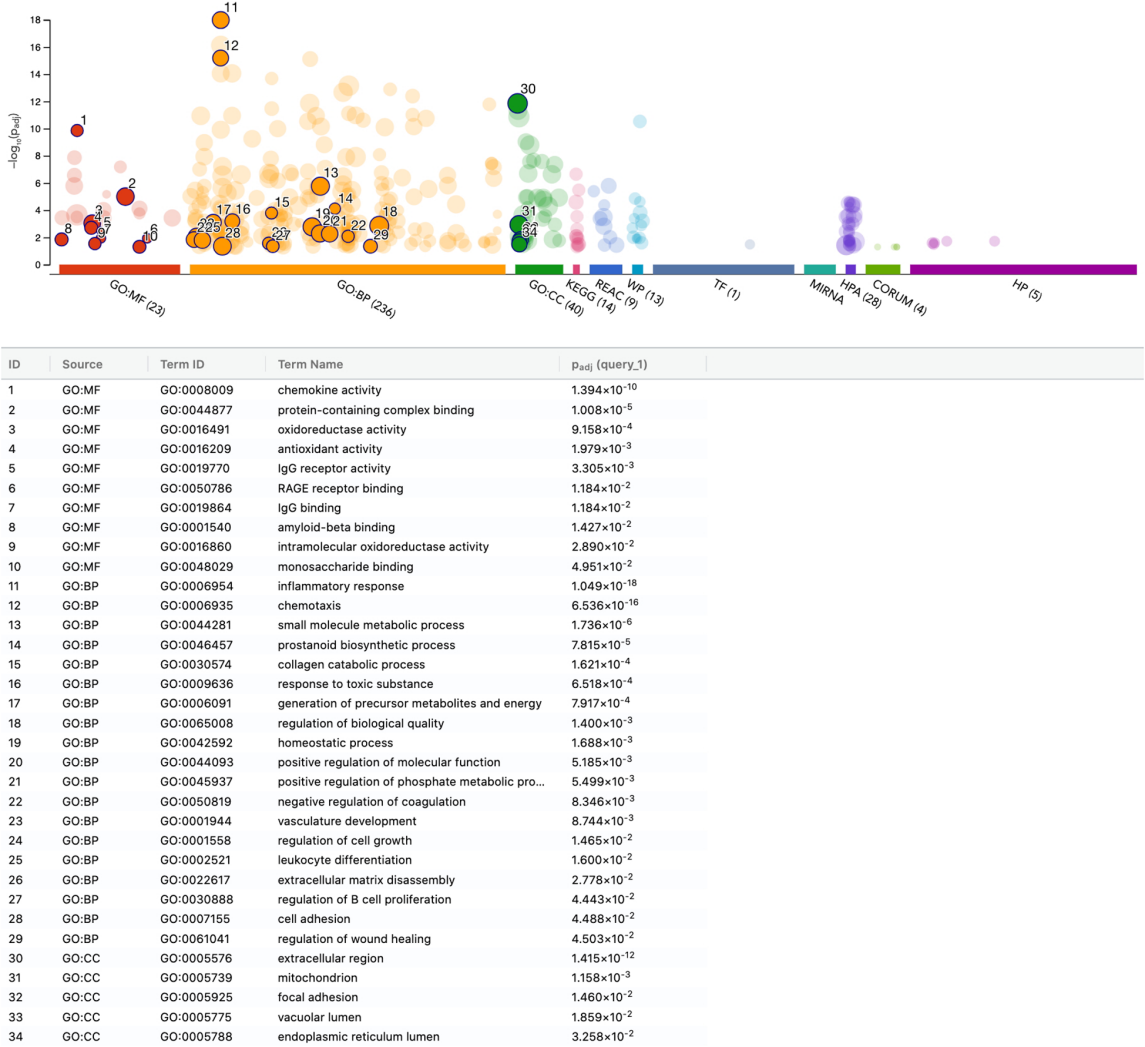
Functional enrichment analysis of upregulated genes in U937 macrophages stimulated with PRF lysates. Functional enrichment analysis, also known as over-representation analysis (ORA), was performed using the g:Profiler web-based platform. The plot depicts the top significantly enriched pathways derived from Gene Ontology and other integrated databases. Pathways are ranked and labeled numerically according to significance. P-values were adjusted for multiple testing using the Benjamini–Hochberg correction method (Padj).

**Figure 6:**
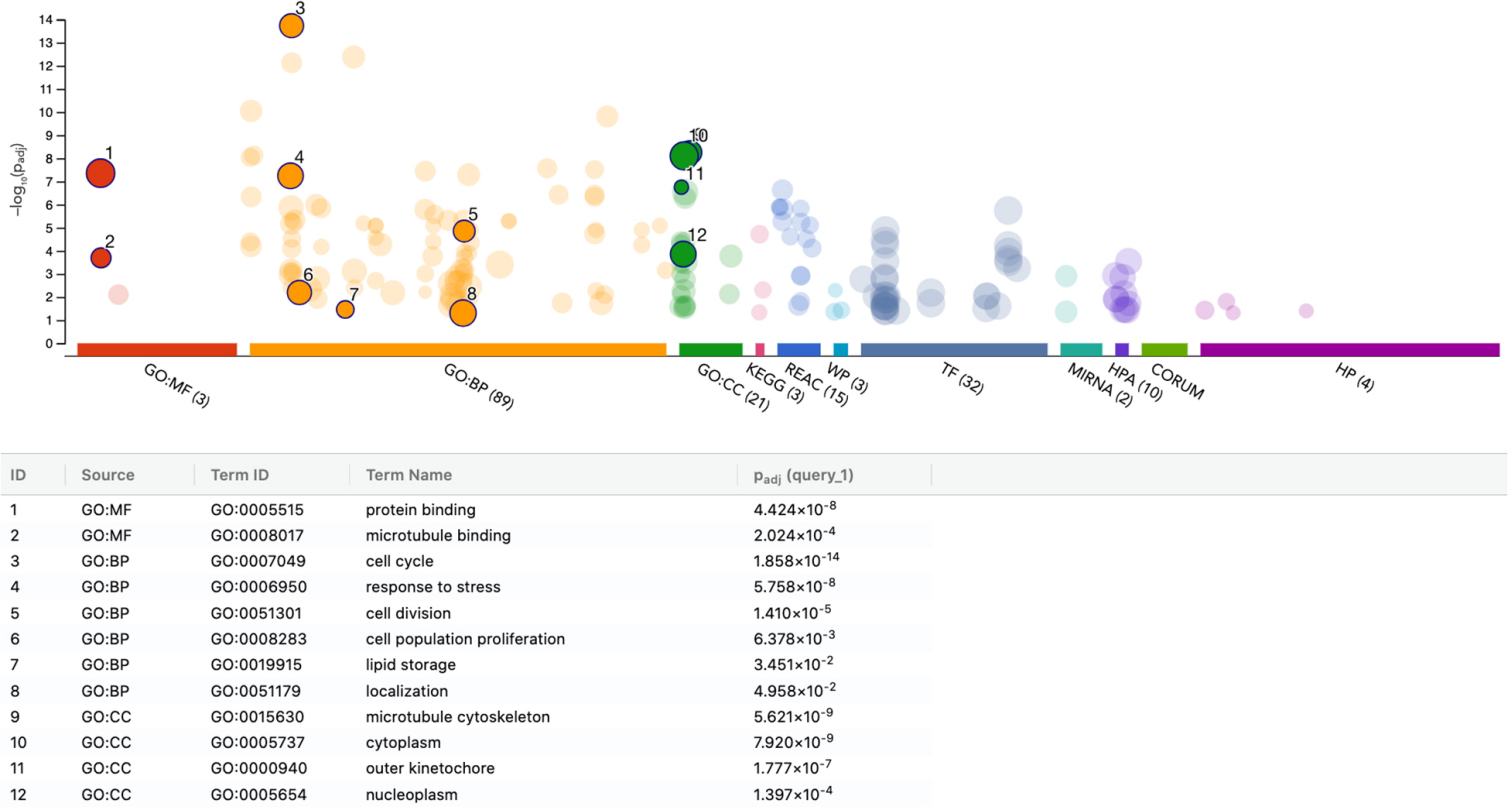
Functional enrichment analysis of downregulated genes in U937 macrophages stimulated with PRF lysates. Functional enrichment analysis (over-representation analysis, ORA) was performed using the g:Profiler web-based platform. The figure displays the top significantly enriched pathways identified from Gene Ontology and other integrated databases, ranked and labeled numerically by significance. p-values were adjusted for multiple testing using the Benjamini–Hochberg correction method (Padj).

Moreover, genes associated with collagen catabolic process (GO:0030574), including CTSL, FAP, MMP3, MMP7, MMP9, MMP12, MMP14, MMP19, and MRC2, were prominently upregulated, indicating extracellular matrix remodeling in response to PRF stimulation. In contrast, downregulated genes were enriched in spindle assembly checkpoint signaling (GO:0071173), featuring AURKB, BUB1, BUB1B, CDC20, CENPF, GEN1, KNL1, NUF2, SPC25, and ZWINT, suggesting suppression of proliferative pathways. Collectively, these findings show that PRF lysates trigger a strong proinflammatory response and matrix remodeling. transcriptional program in U937 macrophages (**Figures 5**, **6**).

**Figure 7:**
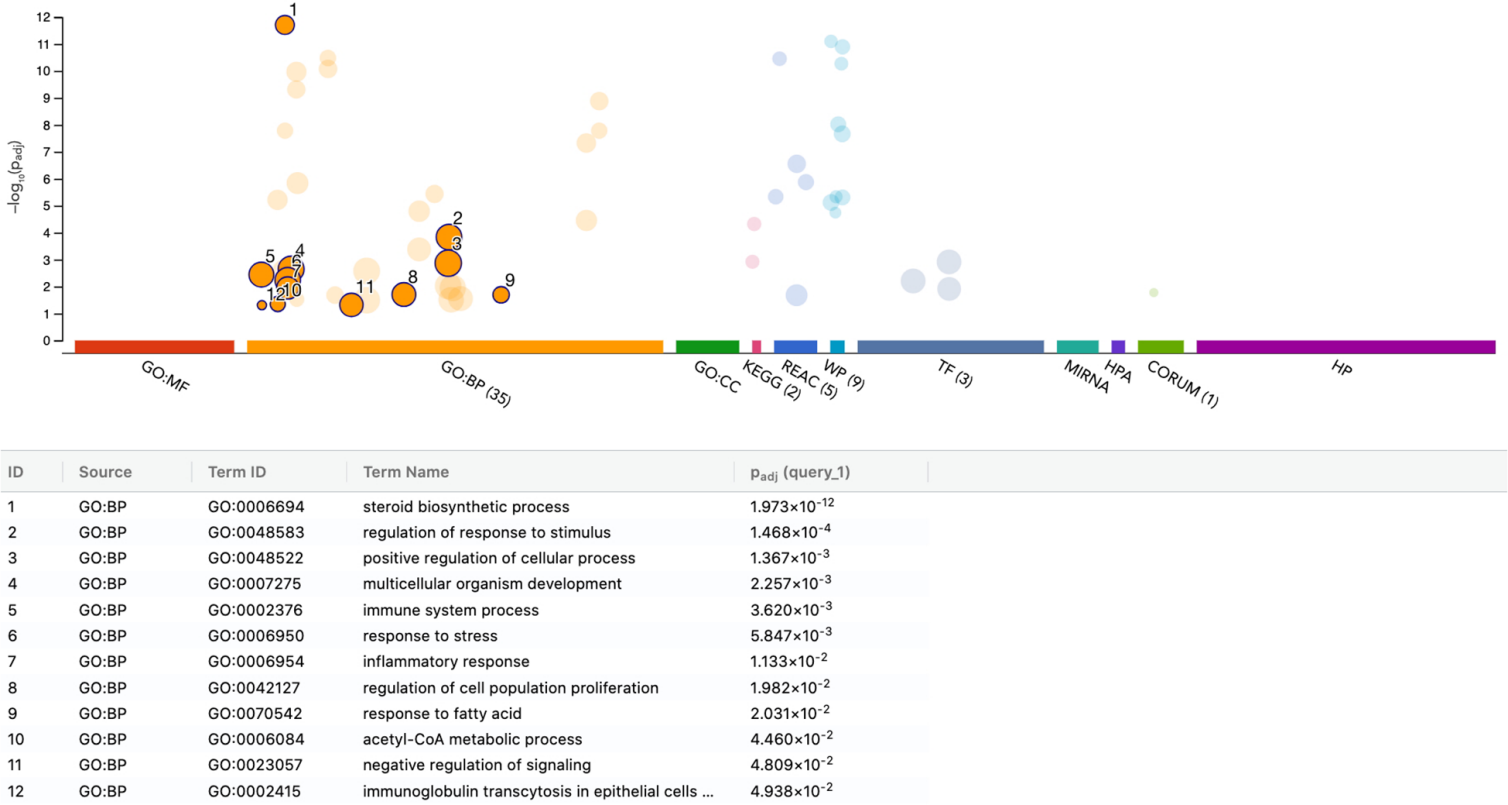
g: functional enrichment analysis of upregulated genes in THP-1 macrophages stimulated with PRF lysates. Functional enrichment analysis (over-representation analysis, ORA) was conducted using the g:Profiler web-based platform. The image displays the top significantly enriched pathways identified from Gene Ontology and related databases, ranked and labeled numerically by significance. p-values were adjusted for multiple testing using the Benjamini–Hochberg correction method (Padj).

**Figure 8:**
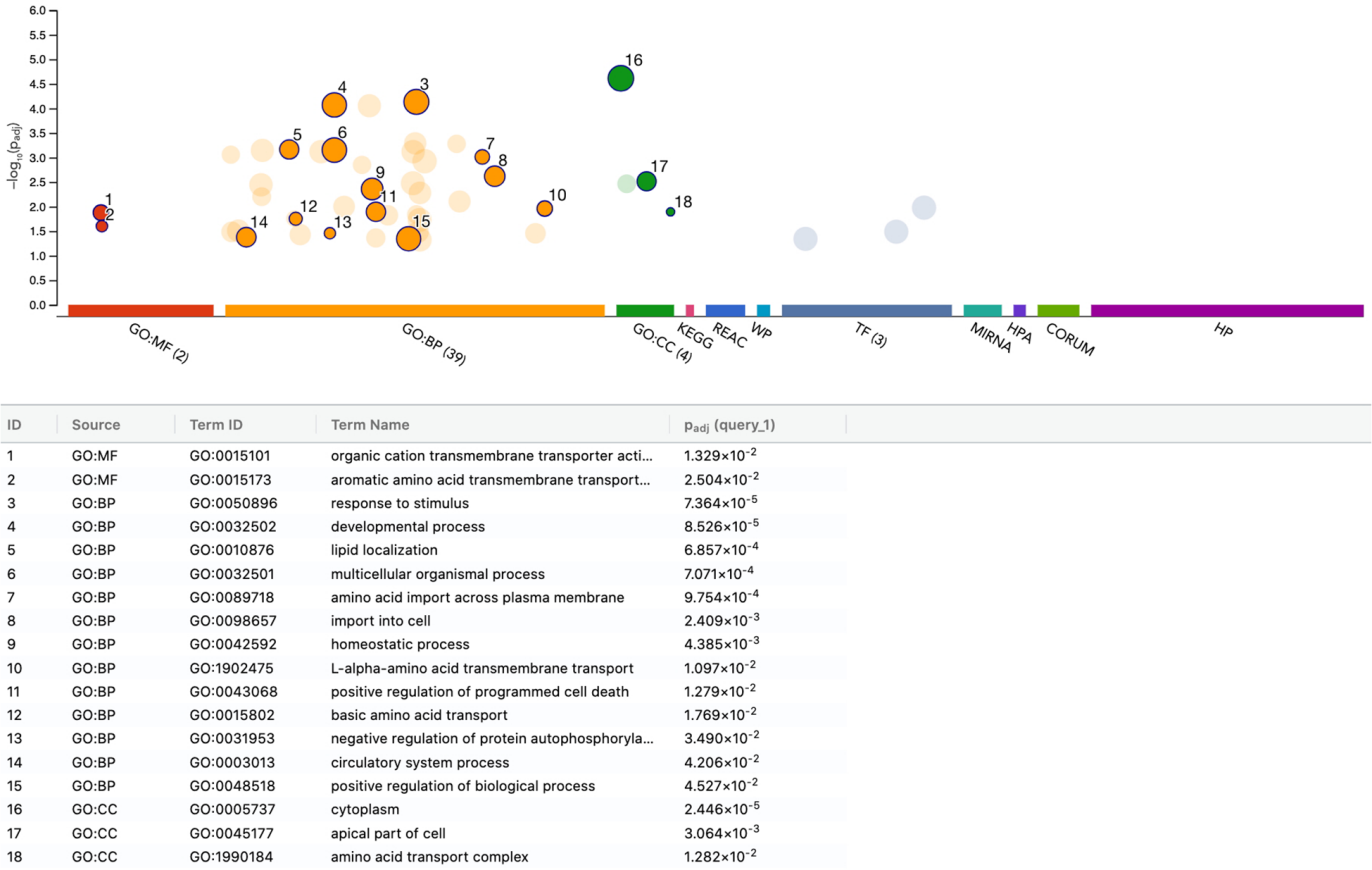
Functional enrichment analysis of downregulated genes in THP-1 macrophages stimulated with PRF lysates. Functional enrichment analysis (over-representation analysis, ORA) was performed using the g:Profiler web-based platform. The figure illustrates the top significantly enriched pathways identified from Gene Ontology and related databases, ranked and labeled numerically by significance. p-values were adjusted for multiple testing using the Benjamini–Hochberg correction method (Padj).

To further characterize the transcriptional response of THP-1 macrophages to PRF lysates, we performed functional enrichment analysis of the 116 upregulated and 149 downregulated genes (adjusted p < 0.05). Among the upregulated genes, enrichment was most pronounced for the steroid biosynthetic process (GO:0006694), encompassing ACLY, BMP6, DHCR7, EGR1, ERG28, FDPS, HMGCS1, HSD17B12, IDI1, INSIG1, MSMO1, MVD, SC5D, SNAI1, SQLE, and STARD4. Conversely, the downregulated gene set was enriched for amino acid import across the plasma membrane (GO:0089718), including RGS2, SLC3A2, SLC6A9, SLC7A1, SLC7A2, and SLC7A5. Taken together, these data indicate that PRF lysates primarily modulate lipid and sterol metabolic pathways in THP-1 macrophages, rather than inducing the pro-inflammatory polarization observed in U937 cells. Thus, the THP-1 response appears more metabolically adaptive than inflammatory (**Figures 7**, **8**).

### RT-PCR analysis of selected genes in U937 cells exposed to PRF lysates

**Figure 9:**
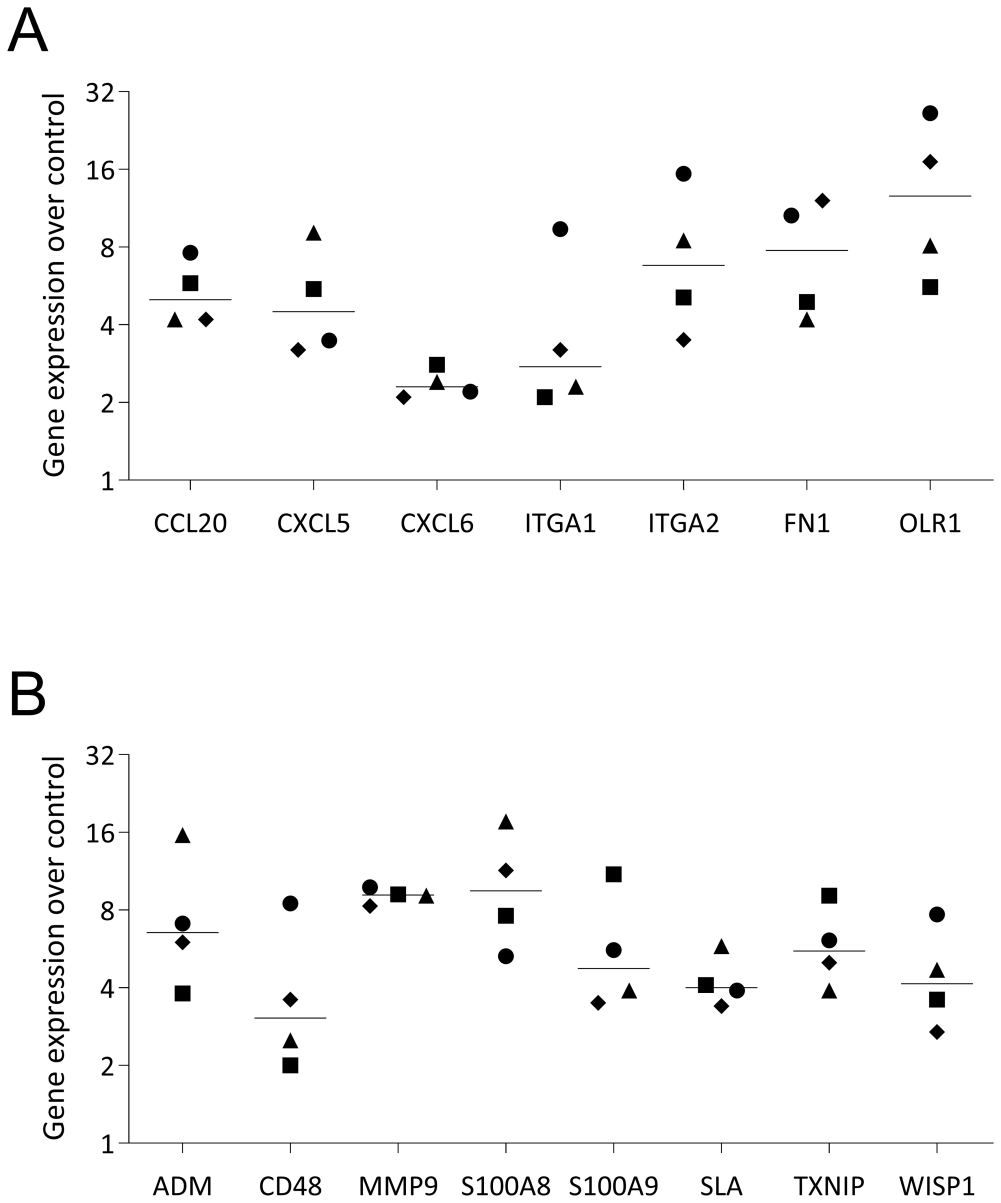
RT–qPCR validation of selected genes in U937 macrophages stimulated with PRF lysates. U937 macrophages were exposed to PRF lysates for 48 hours before quantitative RT–PCR analysis. Data represent the median of four independent experiments and two gene panels **(A, B)**.

**Figure 10:**
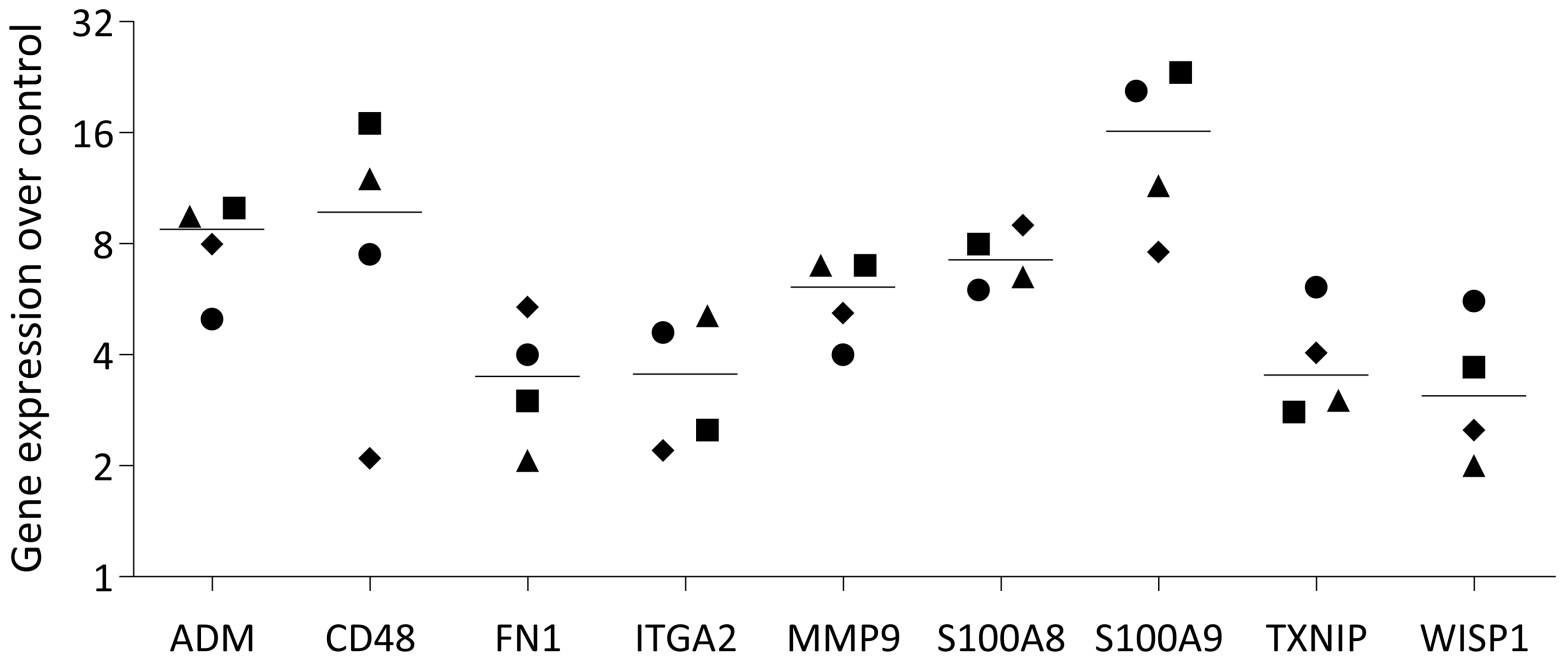
RT–qPCR validation of selected genes in THP-1 macrophages stimulated with PRF lysates. THP-1 macrophages were exposed to PRF lysates for 48 hours before quantitative RT–PCR analysis. Data represent the median from four independent experiments.

**Figure 11:**
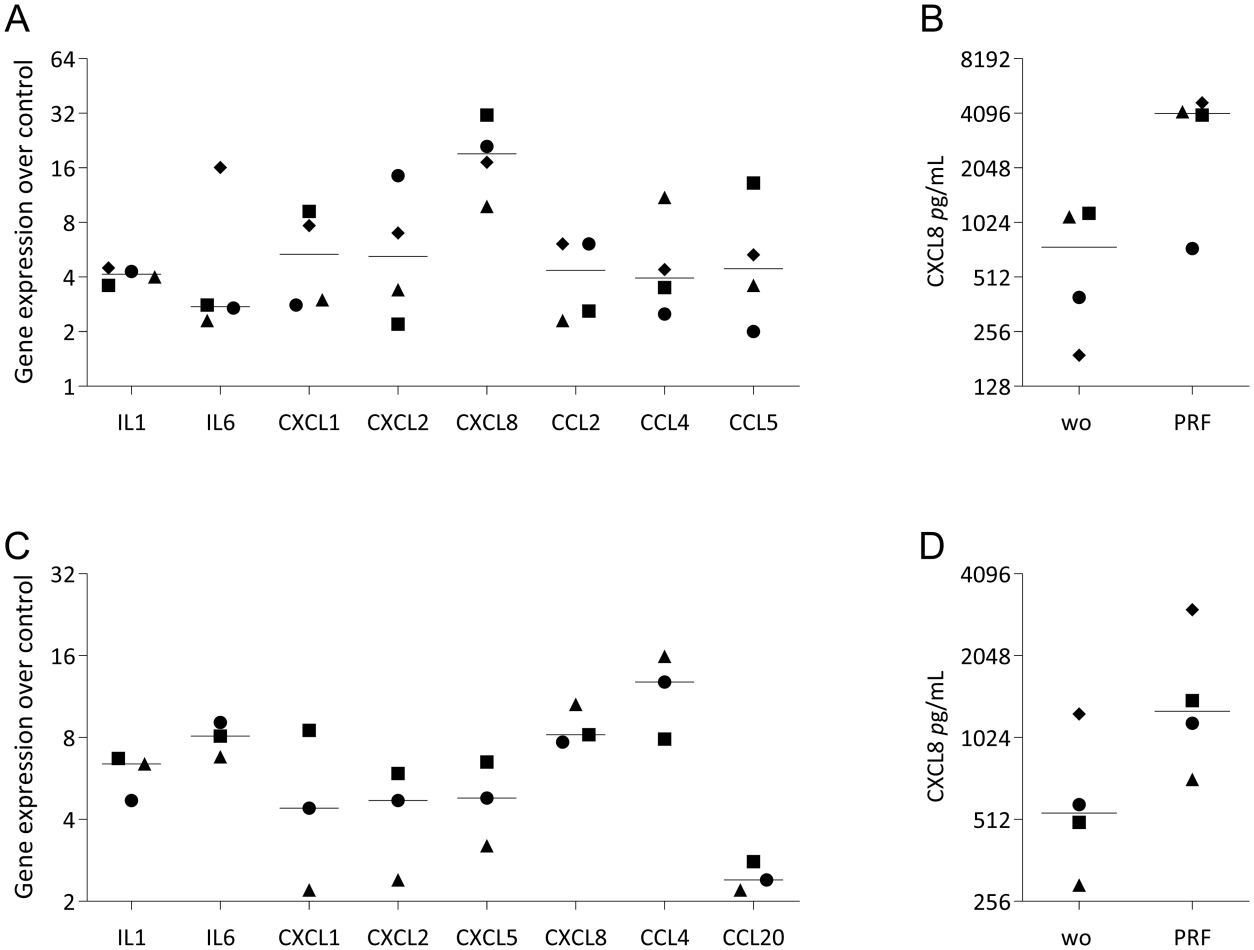
RT–qPCR and ELISA analysis of inflammatory gene expression in U937 and THP-1 macrophages stimulated with PRF lysates. U937 **(A, B)** and THP-1 **(C, D)** macrophages were exposed to PRF lysates for 48 hours before quantitative RT–PCR analysis. ELISA quantified CXCL8 protein levels in the corresponding cell culture supernatants. Data represent the median of independent experiments.

Finally, to validate the RNA sequencing data and reinforce the observation that, at least *in vitro*, PRF elicits a predominantly pro-inflammatory response, we selected a representative panel of genes for verification. In U937 and THP-1 macrophages, PRF exposure induced the expected upregulation of S100A8, S100A9, CD38, ITGA2, ITGA6, and OLR1 (**Figures 9**, **10**), confirming the RNAseq results. Furthermore, **Figure 11** presents a chemokine expression panel regulated by PRF in both U937 and THP-1 macrophages, along with the corresponding protein-level changes, thereby strengthening evidence that PRF stimulation promotes an inflammatory transcriptional and translational profile.

## Discussion

This study was motivated by the need to elucidate how platelet-rich fibrin (PRF)—a widely applied autologous biomaterial in regenerative medicine (18–24, 49), and a physiological derivative of the natural blood clot devoid of erythrocytes (15, 17), modulates macrophage behavior, a cell population pivotal for both wound healing (25–28) and bone regeneration (29, 30). In addition to the accumulation of monocytes within the PRF matrix itself (17), macrophages at the tissue defect site are directly exposed to the bioactive milieu released from PRF, placing them at the center of its immunoregenerative activity. Building upon this premise, our previous work demonstrated that PBMCs respond to PRF lysates with a robust inflammatory transcriptional signature, characterized by the upregulation of human leukocyte antigens, Fcγ receptors, chemokines, calprotectin, complement factors, and interferon-inducible guanylate-binding proteins (34). However, due to the inherent limitations of bulk RNA sequencing, it remained unclear which specific immune cell subsets—such as monocytes, dendritic cells, or lymphocytes—were primarily responsible for these transcriptional changes.

In the present study, we employed U937 and THP-1 cells used as macrophage models to provide cell–type–specific insights into the molecular responses elicited by PRF lysates. The inclusion of THP-1 cells was intentional, as our aim was not only to describe PRF-induced transcriptional changes, but also to identify the more suitable *in vitro* bioassay for PRF research. In this context, the limited inflammatory response of THP-1 cells is not a negative finding, but an important comparative result. While U937 cells showed a robust inflammatory signature, THP-1 cells displayed a weaker and more metabolically oriented response. This side-by-side comparison strengthens our conclusion that U937 cells are more sensitive and therefore more appropriate for studying PRF-induced macrophage activation. This strategy offers a mechanistic framework for identifying potential target cells involved in PRF-mediated immunomodulation. The rationale for this approach is further supported by growing evidence that platelet–monocyte and platelet–macrophage interactions constitute a critical link between coagulation and inflammation (10) and can drive macrophage polarization toward proinflammatory phenotypes (11, 12). Strikingly, strong evidence for the involvement of inflammatory macrophages during early wound healing has emerged from single-cell RNA sequencing (scRNA-seq) analyses of human wound biopsies (14).

Our findings obtained with U937 macrophages closely align with this *in vivo* behavior, reinforcing the translational relevance of our experimental model. In the clinical *in vivo* dataset, four distinct macrophage clusters were identified, including two populations characterized by inflammatory transcriptional signatures. Remarkably, we observed a substantial overlap between our U937 transcriptome and these *in vivo* macrophage subsets—29 genes shared with the “Mac infl” cluster and 22 with “Mac1”—underscoring the biological validity of the PRF-induced signature. This overlap encompasses key gene families associated with inflammatory and tissue-remodeling functions, including chemokines (CCL2, CCL3, CCL4, CCL20, CXCL1, CXCL5, CXCL8), RAGE receptor ligands (S100A8, S100A9), and proteases (MMP-9, MMP-19). Additional shared genes, such as A2M, CD163, CTSL, FABP5, FCGR3A, FN1, IL1RN, KCTD12, LHFPL2, LIPA, OLR1, PLAU, SEMA3C, SERPINB2, SLC11A1, SLC16A10, SPP1, TREM2, and VCAN, further support the notion that PRF activates a macrophage program reflective of early inflammatory wound-healing responses. Collectively, the strong concordance between our U937 transcriptional profile and *in vivo* inflammatory macrophage clusters highlights the clinical relevance of our findings and reinforces the concept that PRF lysates recapitulate key features of early macrophage activation in wound healing (14).

When compared with our previous study using PBMCs, the transcriptional response of U937 macrophages showed remarkable concordance, reinforcing the relevance of this model. Specifically, U937 cells shared 56 upregulated genes with PBMCs, including Fcγ receptors (FCGR1A, FCGR2A, FCGR2B, FCGR3A), chemokines (CCL2, CCL7), calprotectin components (S100A8, S100A9, S100A12), and complement factor (C1QA). Additional shared genes comprised CD38, CD74, CD84, ITGA1, OLR1, MMP14, HBEGF, EGR1, FN1, cathepsins (CTSC, CTSL), and serpins (SERPINA1, SERPINB2). In contrast, THP-1 macrophages exhibited overlap with only 13 upregulated genes, including EGR1, S100A12, SERPINA1, FN1, and IFI6. These findings suggest that the inflammatory transcriptional signature observed in PBMCs is, at least in part, driven by the monocyte-derived macrophage fraction, represented here by U937 cells. Consequently, this study not only corroborates our earlier observations but also advances them by delineating cell-type–specific responses and identifying a robust panel of highly regulated genes. Such a panel may serve as the foundation for establishing a standardized *in vitro* bioassay to evaluate the biological activity of PRF and its derivatives.

Our findings can also be contextualized by comparing them with previous transcriptomic analyses of U937 macrophages stimulated with IFNG and LPS, which activate classical innate immune pathways (42). Notably, we identified 79 genes that were commonly upregulated in U937 cells exposed to either PRF lysates or IFNG/LPS, including chemokines (CCL3, CCL4, CCL5, CCL7, CCL8, CCL20, CXCL5, CXCL6, CXCL8), interleukin signaling components (IL1B, IL1RN), integrin subunits (ITGA1, ITGA2), matrix metalloproteinases (MMP3, MMP9, MMP12, MMP14), metallothioneins (MT1G, MT1X), inflammasome-related genes (NLRP3), prostaglandin synthesis enzymes (PTGES), calprotectin subunits (S100A8, S100A9, S100A12), and serpins (SERPINA1, SERPINB2). However, PRF lysates did not induce the expression of canonical IFNG-driven genes, such as IFIT1–3 and IFIT5, or a panel of cytokines and receptors typically upregulated by IFNG/LPS, including IL1A, IL1R1, IL6, IL15RA, IL18R1, IL19, IL23A, IL24, IL31RA, and IL36G. This divergence indicates that although PRF elicits a proinflammatory transcriptional response, it does not activate classical interferon-γ or Toll-like receptor (TLR)–dependent pathways. Conversely, PRF lysates upregulated a distinct set of genes not responsive to IFNG/LPS in U937 cells, including FCGR1A, FCGR2B, FCGR3A, CCL2, CCL23, CCL26, C1QA, CD38, CD74, CD84, FCRLA, FCRLB, FN1, LILRA1, LILRB1, OLA1, OLR1, PLAU, and PTGS1. Therefore, while there is partial overlap, the molecular profile induced by PRF lysates represents a unique inflammatory program—one that is proinflammatory yet likely operates independently of canonical IFNG/TLR signaling.

To establish a U937-based bioassay for quantifying and comparing the biological activity of PRF and its variants, we leveraged our RNA-seq data together with the *in vivo* inflammatory macrophage signature observed during early human wound healing (14). We propose a focused panel comprising chemokines, calprotectin components (S100A8/S100A9), selected matrix metalloproteinases, and additional inflammation-linked genes (e.g., FN1, OLR1, PLAU) (14). The strong concordance between this U937 signature and *in vivo* inflammatory macrophage clusters underscores its clinical relevance. Notably, the prioritization of chemokines is reinforced by recent bulk RNA-seq showing robust induction of inflammatory genes in gingival fibroblasts exposed to PRF lysates (e.g., CCL2, CCL7, CXCL5, CXCL6, CXCL8, AREG, ITGA2, SERPINB2) (35). Collectively, these observations support a standardized gene panel–based bioassay that not only enables *in vitro* benchmarking of PRF preparations but also provides a tractable framework for interrogating the molecular drivers of the PRF-elicited inflammatory program across U937 macrophages, PBMCs, and— to a lesser extent—fibroblasts.

The proposed U937-based bioassay may facilitate future studies aimed at elucidating the molecular mechanisms underlying the PRF-induced inflammatory response. Several hypotheses merit experimental testing. One possibility is that IL1β, synthesized during platelet activation (50), contributes to the observed macrophage–fibroblast cross-talk, acting as a potent inflammatory stimulus for fibroblasts but not for macrophages (51). Alternatively, PRF lysates may contain interferon-γ (IFNG), which can activate U937 cells [9]. However, platelets do not release IFNG, and the minor fraction derived from neutrophils is unlikely to be functionally relevant (52). Consistent with this, our data indicate that U937 cells do not display a canonical IFNG response.

Another candidate mediator is P-selectin (CD62P), which translocates to the platelet surface and binds to P-selectin glycoprotein ligand-1 (PSGL-1), which is constitutively expressed on U937 cells (53). Yet, this interaction is not considered a classical inflammatory trigger (54). A more plausible mechanism involves CD40 ligand (CD40L), a trimeric member of the TNF superfamily released from activated platelets (55). CD40L can engage CD40, a receptor expressed on various immune and stromal cells (56), and U937 cells can bind soluble CD40L in a CD40-independent manner, modestly increasing CXCL8 expression (57). In contrast, THP-1 cells may lack this response because CD40 expression requires IFNG priming (58). Similarly, gingival fibroblasts, which express CD40, upregulate IL-6 upon CD40 cross-linking (59) and ligation of CD40 by platelet-derived CD40L has been shown to induce CXCL8 and CCL5 in human intestinal fibroblasts and endothelial cells (60, 61). Collectively, these observations support the hypothesis that PRF lysates may exert their pro-inflammatory effects through CD40–CD40L signaling in U937 macrophages. Further investigation using the proposed U937 bioassay could therefore help uncover the clinically relevant pathways by which PRF modulates early inflammatory and regenerative responses.

Although the primary objective of this study was to establish a standardized U937-based bioassay, our findings hold clear clinical relevance. PRF lysates induced a pronounced shift of macrophages toward a proinflammatory phenotype, mirroring the macrophage activation observed during early wound healing (14). This suggests that the proposed bioassay may serve as a valuable platform to investigate the transient activation of inflammatory macrophages that underlie the therapeutic effects of PRF. Importantly, this observation is particularly significant in the context of chronic wounds, which are characterized by a deficient inflammatory phase (14). For instance, venous leg ulcers exhibit a marked depletion of proinflammatory macrophages and a blunted response to chemokines, LPS, and oxidative stress (14). Given the well-documented clinical efficacy of PRF in treating venous ulcers and other non-healing wounds (22, 62–64), our data support the notion that PRF may help reinvigorate macrophage-driven inflammation, thereby reinitiating stalled healing cascades. This transient reactivation of inflammatory macrophages could play a pivotal role in restoring the physiological sequence of wound repair, wherein proinflammatory macrophages and fibroblasts act sequentially to facilitate keratinocyte migration and tissue regeneration (14). Future research should aim to determine the extent to which PRF-induced macrophage activation contributes to the broader regenerative outcomes associated with PRF, potentially extending beyond the evolutionarily conserved functions of the natural blood clot.

## Conclusions

Our findings identify U937 macrophages as a suitable, reproducible bioassay system for investigating inflammatory macrophage polarization in response to PRF exposure. The transcriptional and functional profiles observed closely mirror those of clinical macrophages during early wound healing, underscoring the physiological relevance of this model. Accordingly, the U937-based bioassay provides a powerful platform for studying the recovery and reactivation of inflammatory macrophages in chronic wounds, offering mechanistic insights into PRF’s regenerative potential.

## Data Availability

The datasets presented in this study can be found in online repositories. The names of the repository/repositories and accession number(s) can be found in the article/**Supplementary Material**.

## References

[1] Larsson L Decker AM Nibali L Pilipchuk SP Berglundh T Giannobile WV . Regenerative medicine for periodontal and peri-implant diseases. J Dent Res. (2016) 95:255–66. doi: 10.1177/0022034515618887. PMID: 26608580 PMC4766955

[2] Galarraga-Vinueza ME Barootchi S Nevins ML Nevins M Miron RJ Tavelli L . Twenty-five years of recombinant human growth factors rhpdgf-bb and rhbmp-2 in oral hard and soft tissue regeneration. Periodontol 2000. (2024) 94:483–509. doi: 10.1111/prd.12522. PMID: 37681552

[3] Avila-Ortiz G Ambruster J Barootchi S Chambrone L Chen CY Dixon DR . American Academy of Periodontology best evidence consensus statement on the use of biologics in clinical practice. J Periodontol. (2022) 93:1763–70. doi: 10.1002/JPER.22-0361. PMID: 36279407 PMC10091815

[4] Shanbhag S Mayol M Domic D Bobbili MR Grillari J Sanz M . Harnessing the therapeutic potential of cell secretomes and extracellular vesicles for craniofacial regenerative applications. J Periodontal Res. (2025). doi: 10.1111/jre.70007. PMID: 40642782

[5] Santoro A Voto A Fortino L Guida R Laudisio C Cillo M . Bone defect treatment in regenerative medicine: Exploring natural and synthetic bone substitutes. Int J Mol Sci. (2025) 26:1–26. doi: 10.3390/ijms26073085. PMID: 40243725 PMC11988823

[6] Singer AJ Clark RA . Cutaneous wound healing. N Engl J Med. (1999) 341:738–46. doi: 10.1056/NEJM199909023411006. PMID: 10471461

[7] Hoff P Gaber T Strehl C Schmidt-Bleek K Lang A Huscher D . Immunological characterization of the early human fracture hematoma. Immunol Res. (2016) 64:1195–206. doi: 10.1007/s12026-016-8868-9. PMID: 27629117

[8] Schmidt-Bleek K Kwee BJ Mooney DJ Duda GN . Boon and bane of inflammation in bone tissue regeneration and its link with angiogenesis. Tissue Eng Part B Rev. (2015) 21:354–64. doi: 10.1089/ten.TEB.2014.0677. PMID: 25742724 PMC4533093

[9] Opal SM . Phylogenetic and functional relationships between coagulation and the innate immune response. Crit Care Med. (2000) 28:S77–80. doi: 10.1097/00003246-200009001-00017. PMID: 11007204

[10] Rolling CC Barrett TJ Berger JS . Platelet-monocyte aggregates: Molecular mediators of thromboinflammation. Front Cardiovasc Med. (2023) 10:960398. doi: 10.3389/fcvm.2023.960398. PMID: 37255704 PMC10225702

[11] Passacquale G Vamadevan P Pereira L Hamid C Corrigall V Ferro A . Monocyte-platelet interaction induces a pro-inflammatory phenotype in circulating monocytes. PloS One. (2011) 6:e25595. doi: 10.1371/journal.pone.0025595. PMID: 22022418 PMC3192052

[12] Burbano C Villar-Vesga J Orejuela J Munoz C Vanegas A Vasquez G . Potential involvement of platelet-derived microparticles and microparticles forming immune complexes during monocyte activation in patients with systemic lupus erythematosus. Front Immunol. (2018) 9:322. doi: 10.3389/fimmu.2018.00322. PMID: 29545790 PMC5837989

[13] Boilard E Nigrovic PA Larabee K Watts GF Coblyn JS Weinblatt ME . Platelets amplify inflammation in arthritis via collagen-dependent microparticle production. Science. (2010) 327:580–3. doi: 10.1126/science.1181928. PMID: 20110505 PMC2927861

[14] Liu Z Bian X Luo L Bjorklund AK Li L Zhang L . Spatiotemporal single-cell roadmap of human skin wound healing. Cell Stem Cell. (2025) 32:479–98:e8. doi: 10.1016/j.stem.2024.11.013. PMID: 39729995

[15] Miron RJ Estrin NE Ahmad P Farshidfar N Fujioka-Kobayashi M Zhang Y . Thirty years of autologous platelet concentrates: From platelet-rich plasma to platelet-rich fibrin. J Periodontal Res. (2025). doi: 10.1111/jre.70013. PMID: 40757985

[16] Gruber R . How to explain the beneficial effects of platelet-rich plasma. Periodontol 2000. (2025) 97:95–103. doi: 10.1111/prd.12565. PMID: 38600634 PMC11808461

[17] Miron RJ Fujioka-Kobayashi M Sculean A Zhang Y . Optimization of platelet-rich fibrin. Periodontol 2000. (2024) 94:79–91. doi: 10.1111/prd.12521. PMID: 37681522

[18] Miron RJ Moraschini V Estrin N Shibli JA Cosgarea R Jepsen K . Autogenous platelet concentrates for treatment of intrabony defects-a systematic review with meta-analysis. Periodontol 2000. (2025) 97:153–90. doi: 10.1111/prd.12598. PMID: 39425513 PMC11808470

[19] Miron RJ Moraschini V Estrin NE Shibli JA Cosgarea R Jepsen K . Periodontal regeneration using platelet-rich fibrin. Furcation defects: A systematic review with meta-analysis. Periodontol 2000. (2025) 97:191–214. doi: 10.1111/prd.12583. PMID: 39324633 PMC11808472

[20] Siawasch SAM Yu J Castro AB Temmerman A Teughels W Quirynen M . Autologous platelet concentrates after third molar extraction: A systematic review. Periodontol 2000. (2025) 97:131–52. doi: 10.1111/prd.12600. PMID: 39318055

[21] Siawasch SAM Yu J Castro AB Dhondt R Teughels W Temmerman A . Autologous platelet concentrates in alveolar ridge preservation: A systematic review with meta-analyses. Periodontol 2000. (2025) 97:104–30. doi: 10.1111/prd.12609. PMID: 39345008 PMC11808431

[22] Pinto N Yu J Koirala S Mourao CF Andrade C Rescigno E . L-prf in extra-oral wound care. Periodontol 2000. (2025) 97:342–62. doi: 10.1111/prd.12605. PMID: 39305000 PMC11808448

[23] Narayanaswamy R Patro BP Jeyaraman N Gangadaran P Rajendran RL Nallakumarasamy A . Evolution and clinical advances of platelet-rich fibrin in musculoskeletal regeneration. Bioengineering (Basel). (2023) 10:1–15. doi: 10.3390/bioengineering10010058. PMID: 36671630 PMC9854731

[24] Grecu AF Reclaru L Ardelean LC Nica O Ciuca EM Ciurea ME . Platelet-rich fibrin and its emerging therapeutic benefits for musculoskeletal injury treatment. Med (Kaunas). (2019) 55:1–12. doi: 10.3390/medicina55050141. PMID: 31096718 PMC6572609

[25] Leibovich SJ Ross R . The role of the macrophage in wound repair. A study with hydrocortisone and antimacrophage serum. Am J Pathol. (1975) 78:71–100. 1109560 PMC1915032

[26] Adamson R . Role of macrophages in normal wound healing: An overview. J Wound Care. (2009) 18:349–51. doi: 10.12968/jowc.2009.18.8.43636. PMID: 19862875

[27] Mirza R DiPietro LA Koh TJ . Selective and specific macrophage ablation is detrimental to wound healing in mice. Am J Pathol. (2009) 175:2454–62. doi: 10.2353/ajpath.2009.090248. PMID: 19850888 PMC2789630

[28] Goren I Allmann N Yogev N Schurmann C Linke A Holdener M . A transgenic mouse model of inducible macrophage depletion: Effects of diphtheria toxin-driven lysozyme m-specific cell lineage ablation on wound inflammatory, angiogenic, and contractive processes. Am J Pathol. (2009) 175:132–47. doi: 10.2353/ajpath.2009.081002. PMID: 19528348 PMC2708801

[29] Batoon L Millard SM Wullschleger ME Preda C Wu AC Kaur S . Cd169(+) macrophages are critical for osteoblast maintenance and promote intramembranous and endochondral ossification during bone repair. Biomaterials. (2019) 196:51–66. doi: 10.1016/j.biomaterials.2017.10.033. PMID: 29107337

[30] Batoon L Millard SM Raggatt LJ Pettit AR . Osteomacs and bone regeneration. Curr Osteoporos Rep. (2017) 15:385–95. doi: 10.1007/s11914-017-0384-x. PMID: 28647885

[31] Sordi MB Panahipour L Kargarpour Z Gruber R . Platelet-rich fibrin reduces il-1β release from macrophages undergoing pyroptosis. Int J Mol Sci. (2022) 23:1–15. doi: 10.3390/ijms23158306. PMID: 35955441 PMC9368224

[32] Kargarpour Z Panahipour L Mildner M Miron RJ Gruber R . Lipids of platelet-rich fibrin reduce the inflammatory response in mesenchymal cells and macrophages. Cells. (2023) 12. doi: 10.3390/cells12040634. PMID: 36831301 PMC9954017

[33] Nasirzade J Kargarpour Z Hasannia S Strauss FJ Gruber R . Platelet-rich fibrin elicits an anti-inflammatory response in macrophages *in vitro*. J Periodontol. (2020) 91:244–52. doi: 10.1002/JPER.19-0216. PMID: 31376159 PMC7065136

[34] Panahipour L Kargarpour Z Mildner M Kuhtreiber H Gruber R . Rnaseq of peripheral blood mononucleated cells exposed to platelet-rich fibrin and enamel matrix derivatives. Sci Rep. (2025) 15:3661. doi: 10.1038/s41598-025-86791-5. PMID: 39881164 PMC11779933

[35] Imani A Panahipour L Kuhtreiber H Mildner M Gruber R . Rnaseq of gingival fibroblasts exposed to prf membrane lysates and prf serum. Cells. (2024) 13. doi: 10.3390/cells13151308. PMID: 39120336 PMC11311358

[36] Kargarpour Z Nasirzade J Panahipour L Miron RJ Gruber R . Platelet-rich fibrin decreases the inflammatory response of mesenchymal cells. Int J Mol Sci. (2021) 22:1–11. doi: 10.3390/ijms222111333. PMID: 34768764 PMC8583104

[37] Sundstrom C Nilsson K . Establishment and characterization of a human histiocytic lymphoma cell line (U-937). Int J Cancer. (1976) 17:565–77. doi: 10.1002/ijc.2910170504. PMID: 178611

[38] Tsuchiya S Yamabe M Yamaguchi Y Kobayashi Y Konno T Tada K . Establishment and characterization of a human acute monocytic leukemia cell line (Thp-1). Int J Cancer. (1980) 26:171–6. doi: 10.1002/ijc.2910260208. PMID: 6970727

[39] Chanput W Peters V Wichers H . Thp-1 and U937 cells. In: Verhoeckx K Cotter P Lopez-Exposito I Kleiveland C Lea T Mackie A , editors.The impact of food bioactives on health: *in vitro* and *ex vivo* models. Springer, Cham CH (2015). p. 147–59. Available online at: https://www.ncbi.nlm.nih.gov/books/NBK500159/

[40] Nascimento CR Rodrigues Fernandes NA Gonzalez Maldonado LA Rossa Junior C . Comparison of monocytic cell lines U937 and Thp-1 as macrophage models for *in vitro* studies. Biochem Biophys Rep. (2022) 32:101383. doi: 10.1016/j.bbrep.2022.101383. PMID: 36420419 PMC9677084

[41] Ueno T Yamamoto Y Kawasaki K . Phagocytosis of microparticles increases responsiveness of macrophage-like cell lines U937 and Thp-1 to bacterial lipopolysaccharide and lipopeptide. Sci Rep. (2021) 11:6782. doi: 10.1038/s41598-021-86202-5. PMID: 33762618 PMC7990916

[42] Panahipour L Micucci C Gruber R . Inflammatory response of Thp1 and U937 cells: The rnaseq approach. Cells. (2024) 13:1–16. doi: 10.3390/cells13242062. PMID: 39768153 PMC11674919

[43] Martin M . Cutadapt removes adapter sequences from high-throughput sequencing reads. EMBnetjournal. (2011) 17:10–2. doi: 10.1016/j.ydbio.2015.02.006. PMID: 25732776 PMC4424077

[44] Dobin A Davis CA Schlesinger F Drenkow J Zaleski C Jha S . Star: Ultrafast universal rna-seq aligner. Bioinformatics. (2013) 29:15–21. doi: 10.1093/bioinformatics/bts635. PMID: 23104886 PMC3530905

[45] Love MI Huber W Anders S . Moderated estimation of fold change and dispersion for rna-seq data with deseq2. Genome Biol. (2014) 15:550. doi: 10.1186/s13059-014-0550-8. PMID: 25516281 PMC4302049

[46] Goedhart J Luijsterburg MS . Volcanoser is a web app for creating, exploring, labeling and sharing volcano plots. Sci Rep. (2020) 10:20560. doi: 10.1038/s41598-020-76603-3. PMID: 33239692 PMC7689420

[47] Heberle H Meirelles GV da Silva FR Telles GP Minghim R . Interactivenn: A web-based tool for the analysis of sets through venn diagrams. BMC Bioinf. (2015) 16:169. doi: 10.1186/s12859-015-0611-3. PMID: 25994840 PMC4455604

[48] Kolberg L Raudvere U Kuzmin I Adler P Vilo J Peterson H . G:Profiler-interoperable web service for functional enrichment analysis and gene identifier mapping (2023 update). Nucleic Acids Res. (2023) 51:W207–12. doi: 10.1093/nar/gkad347. PMID: 37144459 PMC10320099

[49] Davies C Miron RJ . Autolougous platelet concentrates in esthetic medicine. Periodontol 2000. (2025) 97:363–419. doi: 10.1111/prd.12582. PMID: 39086171 PMC11808453

[50] Lindemann S Tolley ND Dixon DA McIntyre TM Prescott SM Zimmerman GA . Activated platelets mediate inflammatory signaling by regulated interleukin 1beta synthesis. J Cell Biol. (2001) 154:485–90. doi: 10.1083/jcb.200105058. PMID: 11489912 PMC2196422

[51] Panahipour L Nasserzare S Amer Z Brucke F Stahli A Kreissl A . The anti-inflammatory effect of milk and dairy products on periodontal cells: An *in vitro* approach. Clin Oral Investig. (2019) 23:1959–66. doi: 10.1007/s00784-018-2642-4. PMID: 30238412

[52] Ethuin F Gerard B Benna JE Boutten A Gougereot-Pocidalo MA Jacob L . Human neutrophils produce interferon gamma upon stimulation by interleukin-12. Lab Invest. (2004) 84:1363–71. doi: 10.1038/labinvest.3700148. PMID: 15220936

[53] Hedges EA Hughes AD Liesveld JL King MR . Modulation of selectin-mediated adhesion of flowing lymphoma and bone marrow cells by immobilized sdf-1. Int J Mol Sci. (2014) 15:15061–72. doi: 10.3390/ijms150915061. PMID: 25167133 PMC4200816

[54] Tinoco R Otero DC Takahashi AA Bradley LM . Psgl-1: A new player in the immune checkpoint landscape. Trends Immunol. (2017) 38:323–35. doi: 10.1016/j.it.2017.02.002. PMID: 28262471 PMC5411281

[55] Cognasse F Duchez AC Audoux E Ebermeyer T Arthaud CA Prier A . Platelets as key factors in inflammation: Focus on cd40l/cd40. Front Immunol. (2022) 13:825892. doi: 10.3389/fimmu.2022.825892. PMID: 35185916 PMC8850464

[56] van Kooten C Banchereau J . Cd40-cd40 ligand. J Leukoc Biol. (2000) 67:2–17. doi: 10.1002/jlb.67.1.2. PMID: 10647992

[57] Leveille C Bouillon M Guo W Bolduc J Sharif-Askari E El-Fakhry Y . Cd40 ligand binds to alpha5beta1 integrin and triggers cell signaling. J Biol Chem. (2007) 282:5143–51. doi: 10.1074/jbc.M608342200. PMID: 17182621

[58] Pearson LL Castle BE Kehry MR . Cd40-mediated signaling in monocytic cells: Up-regulation of tumor necrosis factor receptor-associated factor mrnas and activation of mitogen-activated protein kinase signaling pathways. Int Immunol. (2001) 13:273–83. doi: 10.1093/intimm/13.3.273. PMID: 11222496

[59] Sempowski GD Chess PR Moretti AJ Padilla J Phipps RP Blieden TM . Cd40 mediated activation of gingival and periodontal ligament fibroblasts. J Periodontol. (1997) 68:284–92. doi: 10.1902/jop.1997.68.3.284. PMID: 9100204

[60] Vogel JD West GA Danese S De La Motte C Phillips MH Strong SA . Cd40-mediated immune-nonimmune cell interactions induce mucosal fibroblast chemokines leading to t-cell transmigration. Gastroenterology. (2004) 126:63–80. doi: 10.1053/j.gastro.2003.10.046. PMID: 14699489

[61] Danese S de la Motte C Sturm A Vogel JD West GA Strong SA . Platelets trigger a cd40-dependent inflammatory response in the microvasculature of inflammatory bowel disease patients. Gastroenterology. (2003) 124:1249–64. doi: 10.1016/s0016-5085(03)00289-0. PMID: 12730866

[62] Pinto NR Ubilla M Zamora Y Del Rio V Dohan Ehrenfest DM Quirynen M . Leucocyte- and platelet-rich fibrin (l-prf) as a regenerative medicine strategy for the treatment of refractory leg ulcers: A prospective cohort study. Platelets. (2018) 29:468–75. doi: 10.1080/09537104.2017.1327654. PMID: 28727481

[63] Wang F Zhang XL Zhang J Gong S Tao J Xiang H . Therapeutic effectiveness of leukocyte- and platelet-rich fibrin for diabetic foot ulcers: A retrospective study. Curr Med Sci. (2024) 44:568–77. doi: 10.1007/s11596-024-2874-2. PMID: 38789818

[64] Wang Y Wang X Chen R Gu L Liu D Ruan S . The role of leukocyte-platelet-rich fibrin in promoting wound healing in diabetic foot ulcers. Int J Low Extrem Wounds. (2024) 23:306–14. doi: 10.1177/15347346211052811. PMID: 34775872

