# Supplementary material for: Transcriptomic characterization of platelet-rich fibrin-induced macrophage responses identifies U937 cells as a sensitive bioassay
Source: Front Immunol. 2026 Apr 22;17:1722342. doi: 10.3389/fimmu.2026.1722342 (PMC13143749; doi:10.3389/fimmu.2026.1722342)
Supplement: Supplementary file 1 [file DataSheet1.zip › Supplement Files/Supplement Table 1.docx]

The primer sequences.

Genes Forward Sequence Reverse Sequence

ADM ATGAAGCTGGTTTCCGTCG GACATCCGCAGTTCCCTCTT

CCL20 GCTGCTTTGATGTCAGTGCT GCAGTCAAAGTTGCTTGCTG

CCL5 CCTGCTGCTTTGCCTACATTGC ACACACTTGGCGGTTCTTTCGG

CD48 GGCAGGGTCAGACTTGATCC GTAGGTGCTGTTGTCCTCTTTC

CXCL1 TCCTGCATCCCCCATAGTTA CTTCAGGAACAGCCACCAGT

CXCL2 CCCATGGTTAAGAAAATCATCG CTTCAGGAACAGCCACCAAT

CXCL5 AGCTGCGTTGCGTTTGTTTAC TGGCGAACACTTGCAGATTAC

CXCL6 AGAGCTGCGTTGCACTTGTT GCAGTTTACCAATCGTTTTGGGG

CXCL8 AACTTCTCCACAACCCTCTG TTGGCAGCCTTCCTGATTTC

FN1 CGGTGGCTGTCAGTCAAAG AAACCTCGGCTTCCTCCATAA

GAPDH AGCCACATCGCTCAGACAC GCCCAATACGACCAAATCC

IL1 TACCTGTCCTGCGTGTTGAA TCTTTGGGTAATTTTTGGGATCT

IL6 GAAAGGAGACATGTAACAAGAGT GATTTTCACCAGGCAAGTCT

ITGA1 GCTCCTCACTGTTGTTCTACG CGGGCCGCTGAAAGTCATT

ITGA2 GGGAATCAGTATTACACAACGGG CCACAACATCTATGAGGGAAGGG

MMP9 GCCACTACTGTGCCTTTGAGTC CCCTCAGAGAATCGCCAGTACT

OLR1 TTGCCTGGGATTAGTAGTGACC GCTTGCTCTTGTGTTAGGAGGT

S100A8 ATGCCGTCTACAGGGATGAC ACACTCGGTCTCTAGCAATTTCT

S100A9 GGTCATAGAACACATCATGGAGG GGCCTGGCTTATGGTGGTG

SLA CCTGGAGGACCTGGTGAAC TGGTGAGCACACAGCACA

TXNIPv1 ACGCTTCTTCTGGAAGACCA AAGCTCAAAGCCGAACTTGT

WISP1 TGCTGTAAGATGTGCGCTCAG ACACTCCTATTGCGTACCTCG
