# Supplementary figures and images for: Transcriptomic characterization of platelet-rich fibrin-induced macrophage responses identifies U937 cells as a sensitive bioassay
Source: Front Immunol. 2026 Apr 22;17:1722342. doi: 10.3389/fimmu.2026.1722342 (PMC13143749; doi:10.3389/fimmu.2026.1722342)

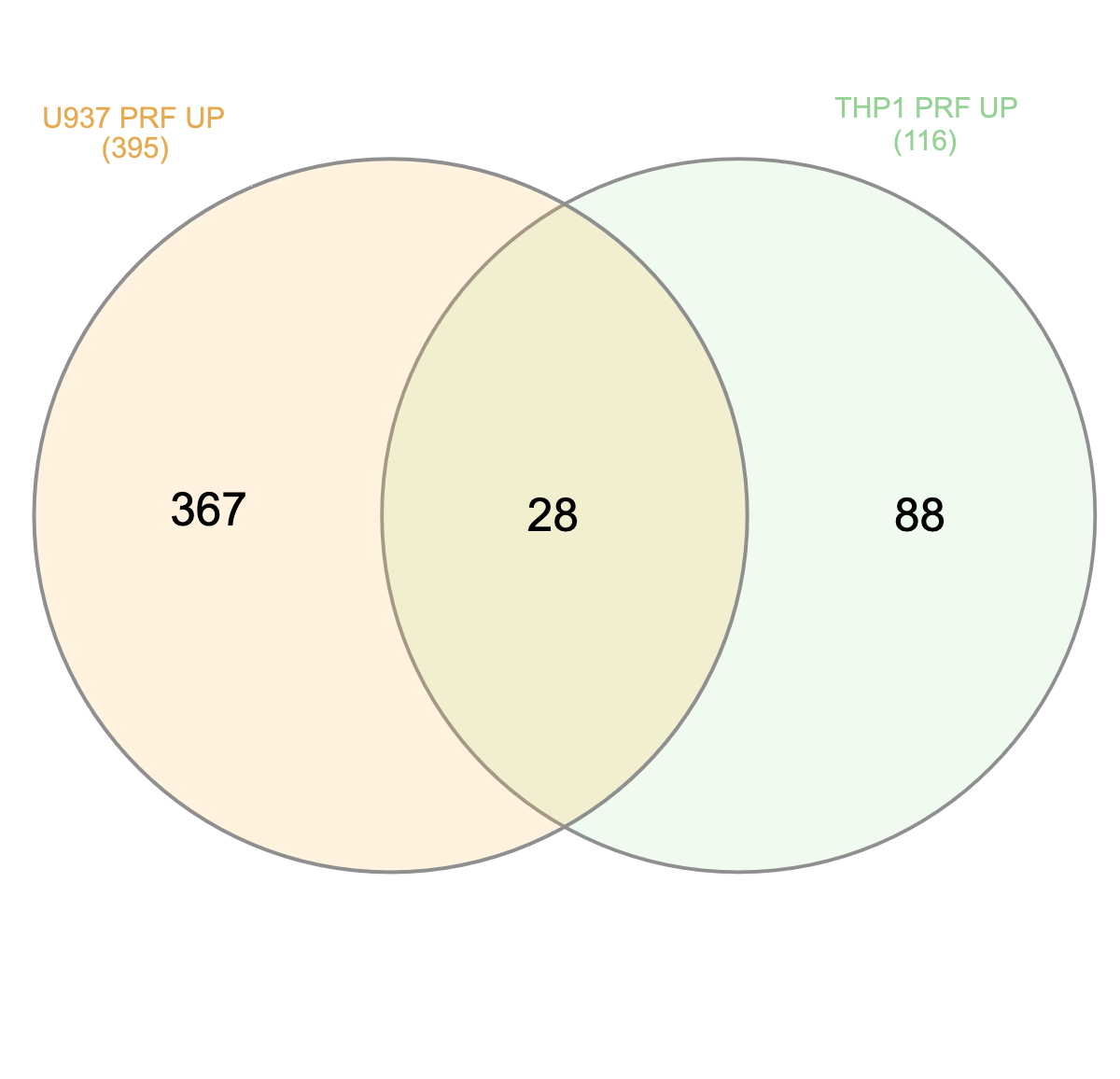

Supplement: Supplementary file 1 [file DataSheet1.zip › Supplement Files/Supplement File 3_Venn Diagram/U937 THP1 PRF Up.png]

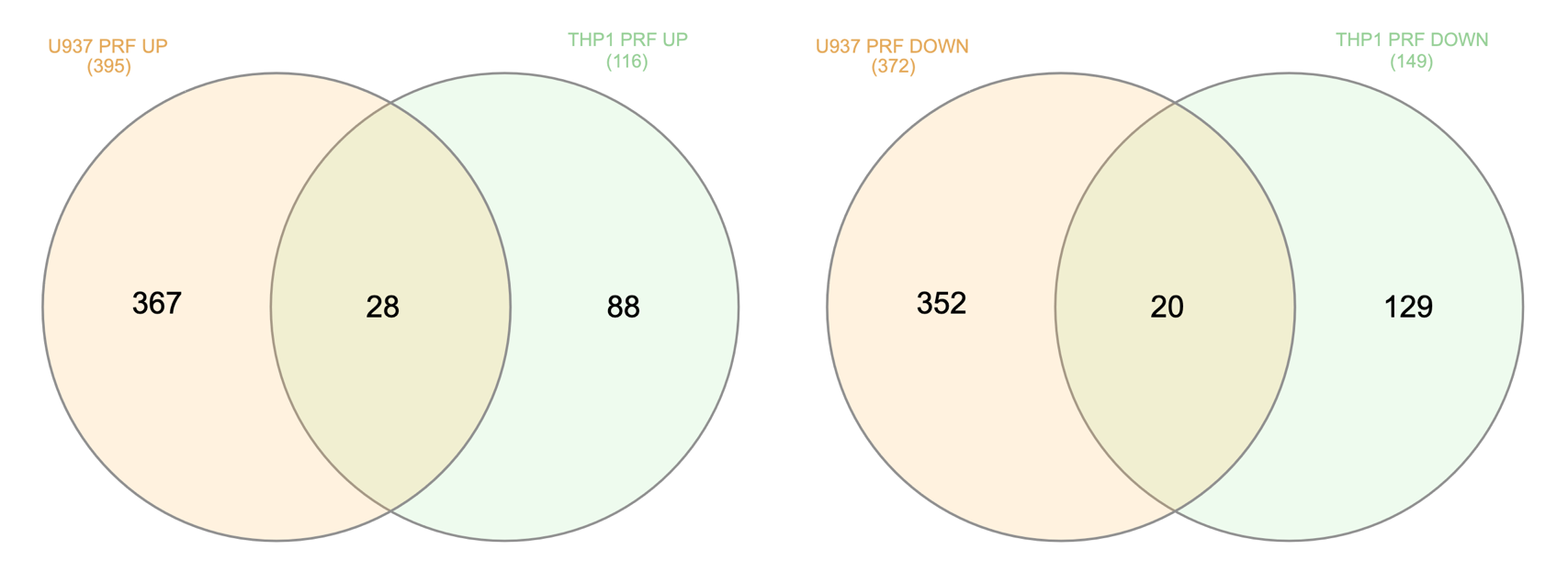

Supplement: Supplementary file 1 [file DataSheet1.zip › Supplement Files/Supplement File 3_Venn Diagram/Ven Figure.png]

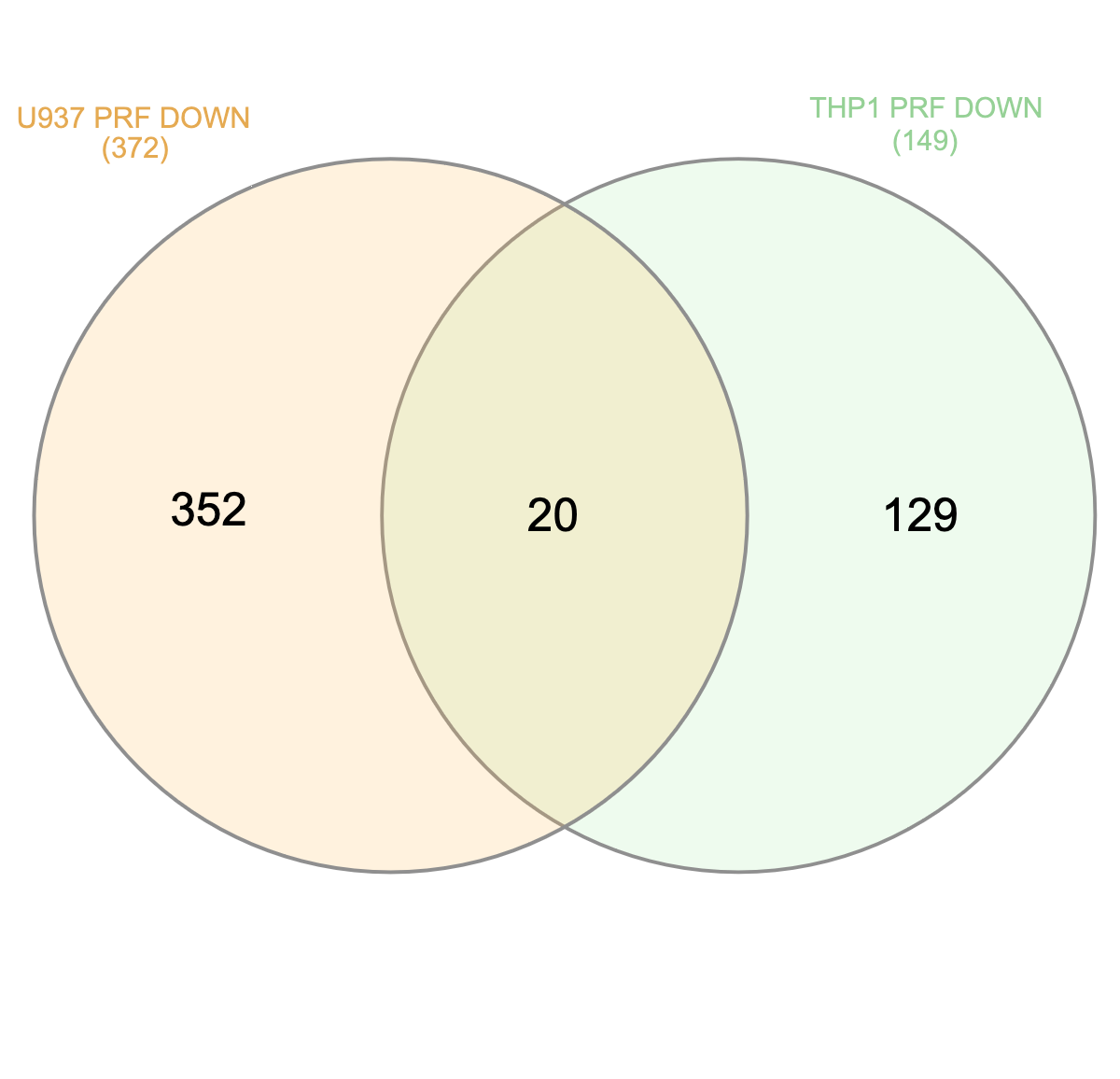

Supplement: Supplementary file 1 [file DataSheet1.zip › Supplement Files/Supplement File 3_Venn Diagram/U937 THP1 PRF Down.png]

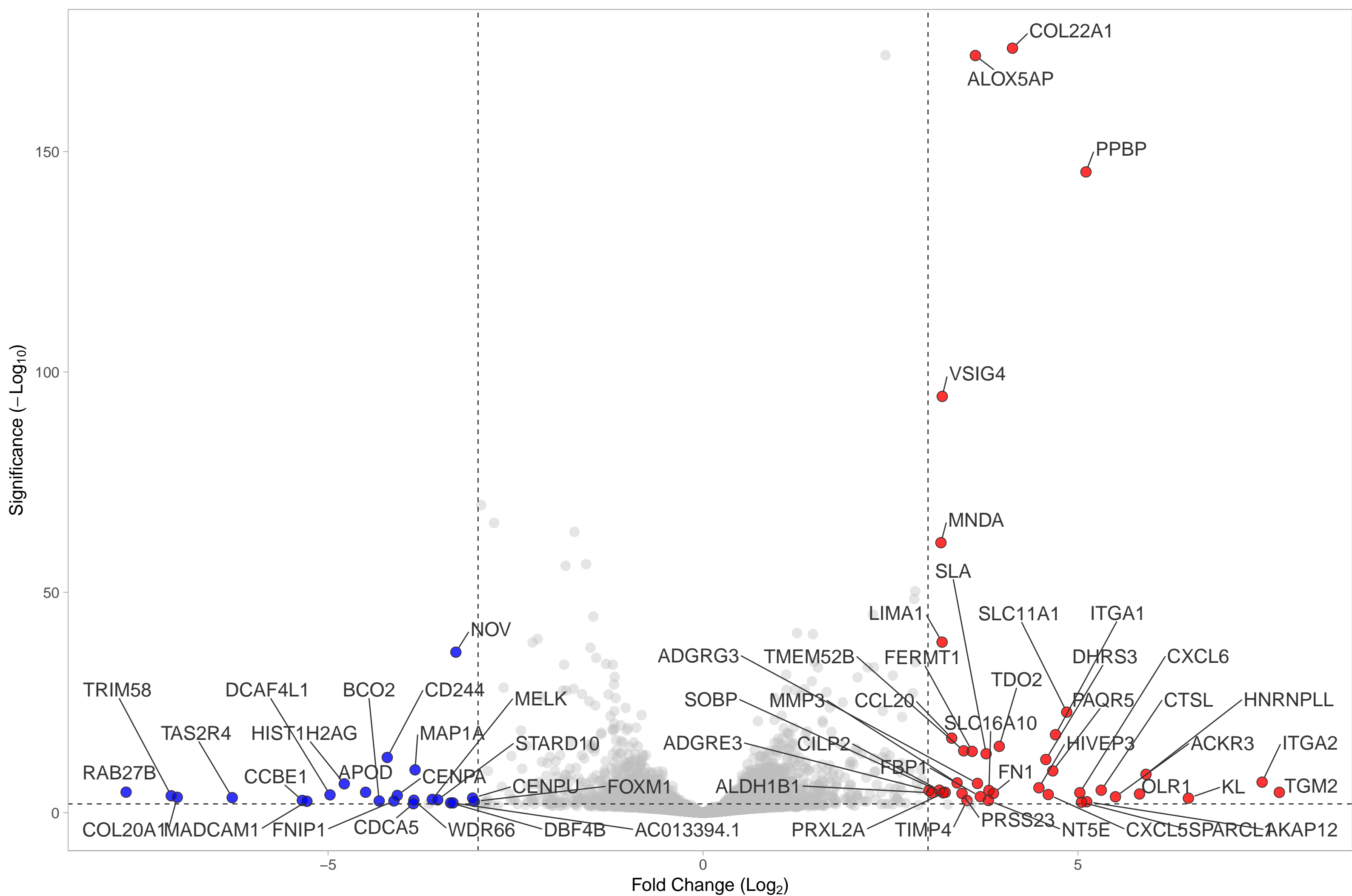

Supplement: Supplementary file 1 [file DataSheet1.zip › Supplement Files/Supplement File 1_Volcano/2.0 -Log10 significance level & 3.0 log2 fold-change/U937.pdf]

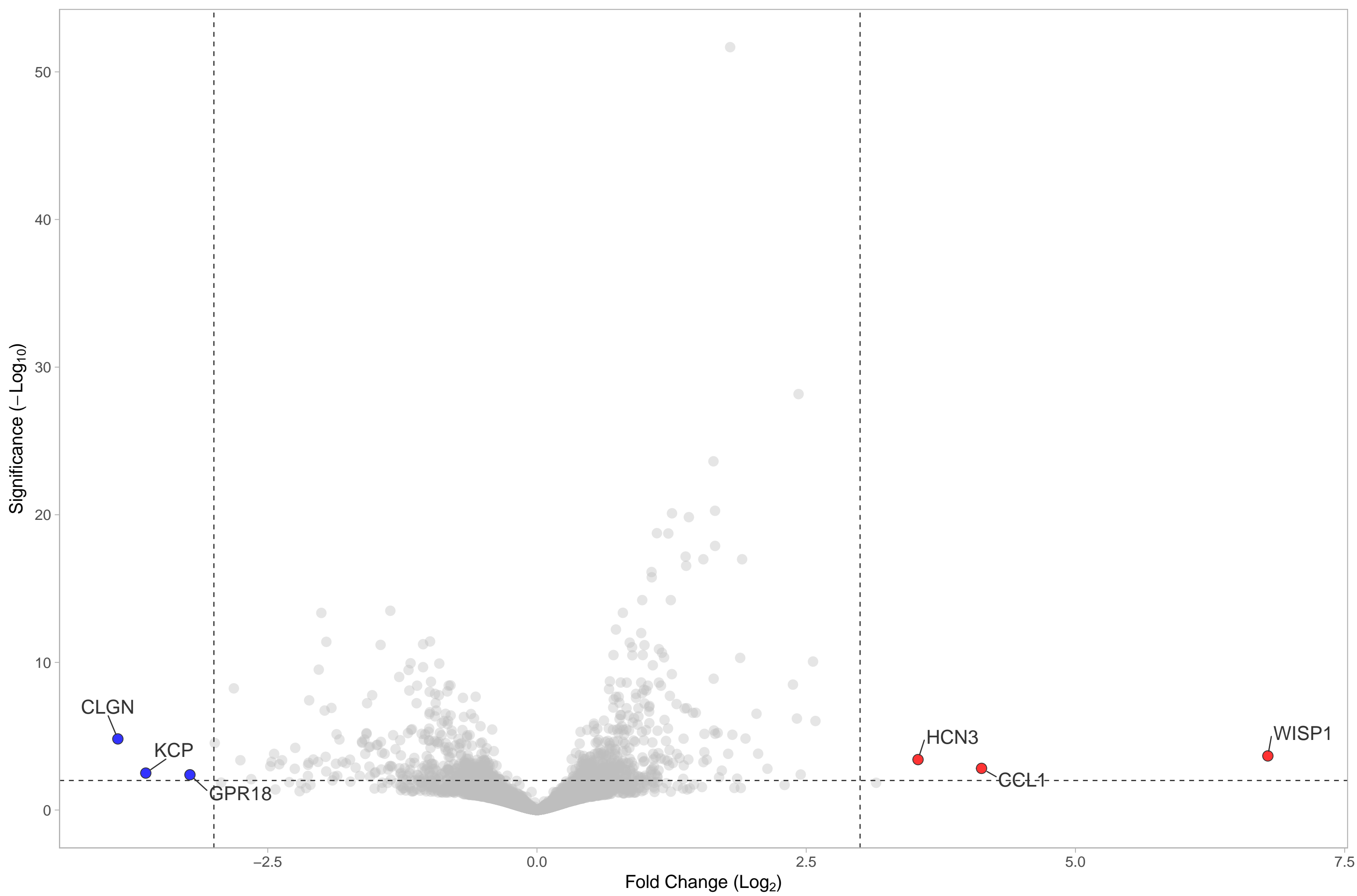

Supplement: Supplementary file 1 [file DataSheet1.zip › Supplement Files/Supplement File 1_Volcano/2.0 -Log10 significance level & 3.0 log2 fold-change/THP1.pdf]
